# Joule Heating-Driven *sp*^2^-C Domains Modulation in Biomass Carbon for High-Performance Bifunctional Oxygen Electrocatalysis

**DOI:** 10.1007/s40820-025-01725-0

**Published:** 2025-04-18

**Authors:** Jiawei He, Yuying Zhao, Yang Li, Qixin Yuan, Yuhan Wu, Kui Wang, Kang Sun, Jingjie Wu, Jianchun Jiang, Baohua Zhang, Liang Wang, Mengmeng Fan

**Affiliations:** 1 Jiangsu Co-Innovation Center of Efficient Processing and Utilization of Forest Resources, International Innovation Center for Forest Chemicals and Materials, College of Chemical Engineering, Nanjing Forestry University, Nanjing, 210037 People’s Republic of China; 2 Institute of Nanochemistry and Nanobiology, School of Environmental and Chemical Engineering, Shanghai University, Shanghai, 200444 People’s Republic of China; 3 Key Lab of Biomass Energy and Material, Jiangsu Province; Jiangsu Co-Innovation Center of Efficient Processing and Utilization of Forest Resources, Institute of Chemical Industry of Forest Products, Chinese Academy of Forestry, Nanjing, 210042 People’s Republic of China; 4 Department of Chemical and Environmental Engineering, University of Cincinnati, Cincinnati, OH 45221 USA; 5 Department of Chemical Engineering, School of Environmental and Chemical Engineering, Shanghai University, Shanghai, 200444 People’s Republic of China

**Keywords:** Natural biomass, Carbon-based catalyst, *sp*^2^-C domains, Joule heating, Oxygen electrocatalysis

## Abstract

Natural biomass-derived carbon material is one promising alternative to traditional graphene-based catalyst for oxygen electrocatalysis. However, their electrocatalytic performance were constrained by the limited modulating strategy. Herein, using N-doped commercial coconut shell-derived activated carbon (AC) as catalyst model, the controllably enhanced *sp*^2^-C domains, through an flash Joule heating process, effectively improve the edge defect density and overall graphitization degree of AC catalyst, which tunes the electronic structure of N configurations and accelerates electron transfer, leading to excellent oxygen reduction reaction performance (half-wave potential of 0.884 V_RHE_, equivalent to commercial 20% Pt/C, with a higher kinetic current density of 5.88 mA cm^−2^) and oxygen evolution reaction activity (overpotential of 295 mV at 10 mA cm^2^). In a Zn-air battery, the catalyst shows outstanding cycle stability (over 1200 h) and a peak power density of 121 mW cm^−2^, surpassing commercial Pt/C and RuO_2_ catalysts. Density functional theory simulation reveals that the enhanced catalytic activity arises from the axial regulation of local *sp*^2^-C domains. This work establishes a robust strategy for *sp*^2^-C domain modulation, offering broad applicability in natural biomass-based carbon catalysts for electrocatalysis.

**Supplementary Information:**

The online version contains supplementary material available at 10.1007/s40820-025-01725-0.

## Highlights

- The flash Joule heating controllably enhanced *sp*^2^-domains content in various N-doped natural biomass-based carbon.
- The axial modulation of *sp*^2^-C domains decreased the charge density of pyridinic N and graphitic N configurations resulting into outstanding oxygen electrocatalysis.
- The assembled Zn-air battery with optimized catalyst achieved an over 1200-h cycle stability with the peak power density of 121 mW cm^−2^, exceeding the commercial Pt/C + RuO_2_ catalysts.

**Supplementary Information:**

The online version contains supplementary material available at 10.1007/s40820-025-01725-0.

## Graphical abstract

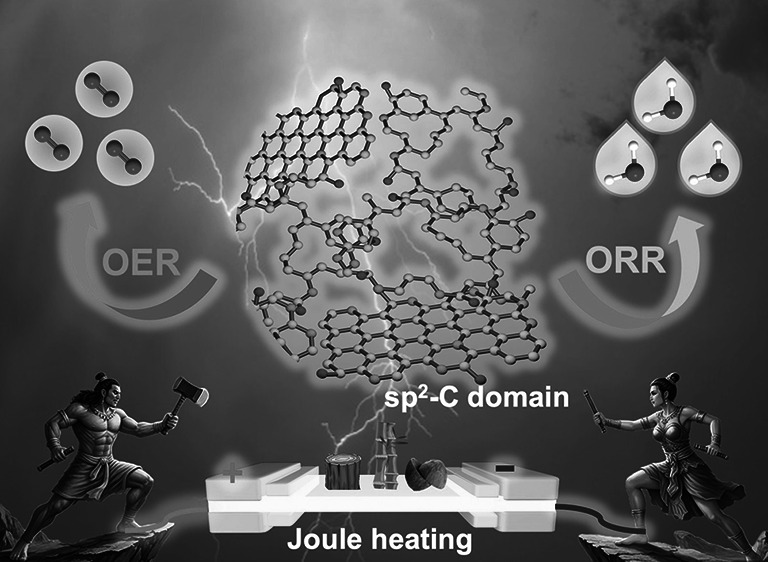

## Introduction

Dimensional carbon materials, such as graphene quantum dots, graphene, and carbon nanotubes, have emerged as highly versatile candidates for electrocatalysis, functioning as active catalysts or catalyst supports [1–3]. Over the past decade, substantial advancements have been achieved in enhancing their catalytic performance through heteroatom doping (e.g., N, S, P, B) and the introduction of geometric carbon defects [4]. These structural and chemical modifications create active, metal-free catalytic sites within the carbon matrix, enabling a synergistic interaction between heteroatom dopants and carbon defects, thereby demonstrating great potential across a variety of electrochemical reactions (e.g., oxygen reduction reaction, CO_2_ reduction reaction) [5, 6]. In addition, compared to traditional metal-based catalysts, metal-free carbon catalysts offer several distinct advantages, including high stability and low resistance [7, 8], making them promising candidates for practical applications. Despite these advancements, the catalytic performance of metal-free carbon materials remains suboptimal, limiting their broader adoption in industrial applications [9, 10].

Achieving high catalytic performance requires a high density of active sites with enhanced intrinsic activity, coupled with efficient electron and mass transfer pathways [11]. Essentially, the design of high-performance carbon catalysts depends on the rational tuning of critical parameters, including the local electronic environment of active sites, the degree of graphitization, and the pore structure [12, 13]. Specifically, to modulate the electronic environment of heteroatom-doped active sites, strategies such as introducing oxygen-containing functional groups or geometric defects like pentagonal carbon rings [14] have been widely employed [15]. These approaches enhance catalytic activity through multiple modulations of heteroatom dopants. Interestingly, heteroatom-doped graphene, enriched with *sp*^2^-C domains [16], consistently outperforms heteroatom-doped amorphous biomass-derived carbon in catalytic applications (e.g., low onset potential, low current density) [17], underscoring the critical role of *sp*^2^-C domains as a critical structural parameter in determining catalytic efficiency. Recent advances in fabrication techniques, such as flash Joule heating, have enabled the rapid synthesis of various nanomaterial like graphene, transition metal chalcogenides, within seconds [18, 19]. This method provides various advantages, including rapid heating to high temperature, high energy utilization, short synthesis time, and low energy consumption. By using these benefits, flash Joule heating serves as a powerful platform for systematically tuning *sp*^2^-C domains and optimizing their catalytic performance [20].

Over the past decade, various biomass-derived molecules (e.g., sucrose, glucose and citric acid) and natural biomass precursors (e.g., lotus leaf, litchi shell and aspen sawdust) have emerged as promising materials for fabricating metal-free carbon catalysts [21–23]. Among these, natural biomass-derived carbon catalysts are particularly attractive due to their low production cost and inherent porous structures [24]. For instance, commercial activated carbon (AC) derived from coconut shells exhibits an exceptionally high specific surface area (3000 m^2^ g^−1^) [25] and abundant mesoporous structures, making it a compelling candidate for industrial applications. If inexpensive natural biomass-derived carbon catalysts, such as ACs, could achieve catalytic performance comparable to that of metal-based catalysts, they could effectively address the cost barriers associated with precious metal or transition metal catalysts [26–28]. While considerable progress has been made in improving the catalytic performance of biomass-based carbon materials through heteroatom doping and defect engineering, the role of *sp*^2^-C domains in these systems remains largely underexplored.

In this study, we employed commercially available coconut shell-derived activated carbon (AC), characterized by its high specific surface area (SSA) and abundant micro/mesoporous structure, as a model platform to fabricate bifunctional oxygen electrocatalysts for oxygen electrocatalysis, encompassing the oxygen reduction reaction (ORR) and oxygen evolution reaction (OER). By utilizing a flash Joule heating process, we significantly enriched the *sp*^2^-C domain content in nitrogen-doped defective AC (N-C_D_), while preserving its high SSA and porous architecture. The synergistic modulation of pyridinic N and graphitic N configurations by local *sp*^2^-C domains resulted in an exceptional ORR half-wave potential (*E*_1/2_) of 0.884 *V*_RHE_ in 0.1 M KOH, as well as an improved OER activity with an overpotential of only 295 mV at a current density of 10 mA cm^−2^, rivaling the performance of commercial Pt/C and RuO_2_ catalysts, respectively. Remarkably, when integrated into a Zn-air battery as an air electrode, the catalyst demonstrated exceptional long-term charge–discharge stability, maintaining performance for over 1200 h. Density functional theory (DFT) calculations revealed that *sp*^2^-C domain can axially modulate the electronic states of active carbon sites, providing mechanistic insights into the enhanced catalytic activity. These findings underscore the critical role of *sp*^2^-C domain modulation in tuning the electronic environment of active sites, offering a versatile and scalable strategy for advancing biomass-derived carbon catalysts in oxygen electrocatalysis.

## Experimental Methods

### Chemicals

Sodium chloride (NaCl, 99.5%) and 2,6-Diaminopyridine (C_5_H_7_N_3_, 99.5%) were purchased from Macklin. Zinc chloride (ZnCl_2_, 98%) was obtained from Energy Chemical. The coconut shell-derived AC was purchased from the MuLinSen activated carbon Jiangsu Co., Ltd. Before use in our study, the AC was washed many times with 0.1 M HCl and deionized (DI) water to remove any impurities.

### Synthesis of Catalysts of Pure C, Pure C_D_, N–C, N-C_D_′ and N-C_D_

For preparing N-C_D_ catalyst, 1.0 g of coconut shell activated carbon, 1.0 g of 2,6-diaminopyridine, 5.0 g of NaCl, and 0.5 g of ZnCl_2_ was first dissolved into 100 mL of DI water, and then the mixture solution was stirred at 100 °C for 12 h to evaporate the water. The dried mixture was annealed at a rate of 5 °C min^−1^ for 2 h at 1000 °C under N_2_ atmosphere (40 sccm). After natural cooling to room temperature, the material was subjected to multiple ultrasonic acid washes using 0.5 M H_2_SO_4_ to remove residual metal salts. The material was then washed to neutral with DI water and dried in an 80 °C vacuum oven to obtain the N-C_D_′ catalyst. The N-C_D_′ was placed between two conductive carbon felts and treated with a flash Joule heating device under Ar atmosphere with the rate of 800 °C s^−1^ for 1s to obtain the N-C_D_ catalyst. Apart from no adding 2, 6-diaminopyridine, NaCl and ZnCl_2_, the preparing conditions of pure C are same to N-C_D_. Apart from no adding 2, 6-diaminopyridine, the preparing conditions of pure C_D_ are same to N-C_D_. Apart from no adding NaCl and ZnCl_2_, the preparing conditions of N–C are same to N-C_D_. Synthesis of N-C_D_′-x through a tube furnace: for preparing N-C_D_′-x (*x* = 1, 30) catalyst, the N-C_D_′ catalysts were further annealed in a tubular furnace at 800 °C, at the heating rate of 10 °C min^−1^, AR atmosphere for 1 and 30 min, respectively.

### Materials Characterization

The morphology characterization information was collected by high-resolution transmission electron microscopy (HR-TEM) (FEI Talos F200s) with energy-dispersive X-ray spectroscopy (XPS, FEI Super-X EDS Detector). The structure and composition characterizations were completed by X-ray photoelectron spectra (Thermo Scientific Al K_α_), X-ray diffractometer (Bruker, D8 FOCUS) equipped with a Cu K_α_ radiation source (*λ* = 0.154 nm), FT-IR (Nicolet IS10). The Raman spectra were obtained by a Renishaw inVia with a 532 nm laser source. The surface area and meso/macropore size distributions were measured by the Brunauer–Emmett–Teller (BET) and Barret-Joyner-Halenda (BJH) methods, respectively. N_2_ adsorption–desorption isotherm tests were performed on a gas adsorption analyzer (ASAP2460, Micromeritics). The XANES measurement were conducted in National synchrotron radiation laboratory (NSRL) in total electron yield (TEY) mode monitoring total current with the base pressure of ∼2 × 10^−10^ mbar in the UHV chamber at throughout the measurement.

### Electrochemical Characterizations

The electrochemical activities of the samples for electrocatalytic ORR and OER performance were performed with a CHI 760E electrochemical workstation in a standard three-electrode system. A graphite rod and an Hg/HgO electrode acted as the counter electrode and reference electrode, respectively. The working electrode used was a glassy carbon rotating disk electrode (RDE) and a rotating ring disk electrode (RRDE) (Pine, USA). The surface areas of the glassy carbon electrode on RDE and RRDE were measured to be 0.19625 and 0.2475 cm^2^, respectively. The catalyst of 5.0 mg was dispersed into the solution of 1 mL (isopropanol: DI water: 5 wt% Nafion solution = 80%: 10%: 10% v/v). The above ink was sonicated for 2 h. And 10, 30, 50, and 60 μL catalyst ink (corresponding catalyst loading: 0.255, 0.765, 1.275, and 1.530 mg cm^−2^, respectively) was deposited onto the RDE (5 mm in diameter) and dried at room temperature naturally. For comparison, commercial Pt/C catalysts were used as a reference for ORR, whereas RuO_2_ catalysts were used as a reference for OER.

### ORR Characterization with RDE

Both cyclic voltammetry (CV) and linear sweep voltammetry (LSV) were used to analyze the ORR activities of the catalysts with O_2_-saturated 0.1 M KOH solution as the electrolyte. Before the RDE test, the electrode was scanned four times at scan rates of 50 and 100 mV s^−1^, respectively, to activate the catalyst. The O_2_ or Ar flow was constant 100 sccm during the RDE or CV test in 0.1 M KOH.

The CV curves were measured at 10 mV s^−1^ with the potential from 0–1.1 V vs. RHE. The LSV performed with a potential from 0–1.1 V vs. RHE at a scan rate of 10 mV s^−1^ with different rotating speeds (400, 625, 900, 1225, 1600, and 2025 rpm). The catalytic stability was measured at 0.45 V vs. RHE for 11 h in 0.1 M KOH. The accelerate durability testing (ADT) involved 5000 cycles of cyclic voltammetry at a scan rate of 100 mV s^−1^. The resistant methanol was measured in 0.1 M KOH at 10 mV s^−1^. The RHE was calculated based on the follow equation.

1 $$V_{{{\text{RHE}}}} = \, V_{{({\text{Hg}}/{\text{HgO}})}} + \, 0.0{592} \times {\text{pH }} + \, 0.0{95}$$

Calibration of Hg/HgO reference electrode with respect to reversible hydrogen electrode (RHE) in H_2_-saturated 0.1 M KOH.

2 $$V_{{({\text{RHE}})}} = \, V_{{({\text{Hg}}/{\text{HgO}})}} + \, 0.{\text{884 V}}$$

The number of electrons transferred (n) during ORR was calculated by Koutecky-Levich (K-L) equation according the LSV curves with varying rotating speed from 400 to 2025 rpm.

3 $$\frac{1}{J} = \frac{1}{{B\omega^{1/2} }} + \frac{1}{{J_{K} }}$$

4 $$B = 0.62\,n\,FC_{0} \left( {D_{0} } \right)^{2/3} Y^{ - 1/6}$$

where ***J*** is current density, ***ω*** is electrode rotation rate, **n** is the overall number of electron transfer, **F** is the Faraday constant (**F** = 96 485 C mol^−1^), ***C***_**0**_ is the bulk concentration of O_2_ (***C***_**0**_ = 1.2 × 10^−3^ mol L^−1^), ***D***_**0**_ is the diffusion coefficient of O_2_ (***D***_**0**_ = 1.9 × 10^−5^ cm s^−1^), and **γ** is the kinematic viscosity of the electrolyte (**γ** = 0.1 m^2^ s^−1^).

The EIS measurements were conducted for the working electrode in a frequency range of 100 kHz to 0.01 Hz with ac perturbation of 5 mV. The EIS data were analyzed using Nyquist plots. The ECSAs were calculated by CV curves in a potential window of 0.95–1.15 V vs. RHE at different scan rates in Ar-saturated 0.1 M KOH.

The curves of resistivity-pressure were measured by resistivity of the powder tester (ST-2722, Suzhou Jingge Electronic Co., Ltd.) which is the combination of four-probe resistivity tester and automatic powder flaker. Different power was filled in a cylindrical shape mold with the diameter of 1.0 cm and height of 0.3 cm, and then different pressure was applied and the corresponding resistivity was recorded.

### ORR Characterization with RRDE

The catalytic performance of the catalysts toward ORR was evaluated based on LSV curves at a scan rate of 10 mV s^−1^ on a RRDE. The yield of hydrogen peroxide and electron transfer number (n) were calculated according to the following equations:

5 $${{H}_2}{{O}_2}\,\,selectivity\text{ \%}=200\frac{{I}_{R}/N}{{I}_{D}+({I}_{R}/N)}$$

6 $$\text{n}=4\frac{{I}_{D}}{{I}_{D}+({I}_{R}/N)}$$

Where *I*_D_ is the disk currents, *I*_R_ is the ring currents, N is the ring current collection efficiency (37% the RRDE electrode: PINE E7R9).

### OER Measurement

The OER test was conducted in an H-cell with 1.0 M KOH electrolyte (pH = 14), reference electrode of Hg/HgO, Pt sheet as counter electrode, and the carbon paper with a catalyst loading of 2 mg cm^−2^ as the working electrode. Obtain LSV curve from high potential to low potential in O_2_-saturated 1.0 M KOH at a scanning rate of 10 mV s^−1^.

### Preparation and Measurements of Zinc–Air Battery (ZAB)

A polished high-purity zinc plate serves as the anode, the electrode composite substrate made of carbon paper, waterproof layer (PTFE + Carbon) and collector layer (nickel foam) is used as the support material of the catalyst, the loading of catalyst on carbon paper is set to 1 mg cm^−2^, serving as the air cathode. For purposes of comparison, a precious metal catalyst comprising Pt/C and RuO_2_ was used (m_Pt/C_:m_RuO2_ = 1:1). The electrolyte solution consisted of KOH (6.0 M) and Zn(OAc)_2_ (0.2 M). All testing procedures were conducted under standard room temperature and atmospheric pressure conditions. The ZABs tests were measured via LAND CT3002A. Charging and discharging times in the stability test were 10 min, and the current density is 5 mA cm^−2^.

### DFT Simulation

The DFT calculations were performed using Vienna ab initio simulation package (VASP) [29]. The electronic exchange–correlation potential was calculated using the Perdew-Burke-Ernzerhof (PBE) functional of generalized gradient approximation (GGA) were used [30–32]. The kinetic energy cutoff was set to 500 eV for the plane-wave basis set. The K-point sampling was obtained from the Monkhorst − Pack scheme with a (3 × 3 × 1) mesh for optimization. The tolerance of the self-consistent field (SCF) convergence was 1.0 × e^−5^ eV, and the max force, stress and displacement were 0.02 eV Å^−1^, 0.05 GPa and 0.001 Å, respectively. The vacuum layer was set around 20 Å to avoid the interaction along z-direction. After relaxation, the distance between two layers was ∼2.0 Å, which implies weak van der Waals interaction between each layer.

The potential-dependence of reaction free energies in the elementary steps involving proton-electron transfers were evaluated using the computational hydrogen electrode (CHE) approach [33].

The Gibbs free energy was calculated using:

7 $$\Delta G = \Delta E_{{\text{n}}} + \Delta ZPE{-}{\text{T}}\Delta S$$

where Δ*G* is Gibbs free energy, Δ*E*_n_ is energy calculate from DFT, S is entropy, T is temperature (298.15 K), and *ZPE* is zero-point energy.

## Results and Discussion

### Characterization of Morphology and Structure

**Fig. 1:**
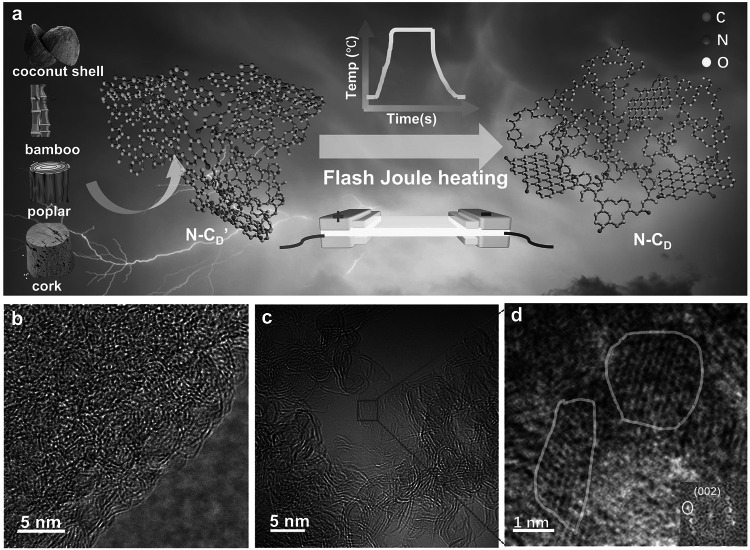
**a** Schematic illustration of N-C_D_ synthesis by Joule heating N-C_D_′ and many different natural biomasses were used to prepare N-C_D_′. **b** High resolution transmission electron microscopy (HR-TEM) image of N-C_D_′. **c** ACTEM image of N-C_D_. **d** Enlarged images from the red square in (**c**), the yellow line area represents *sp*^2^-C domains with regular hexagon structures (inset, FFT pattern for the yellow area in (**c**))

The precursor coconut shell-derived AC with amorphous carbon structure exhibits a high specific surface area (SSA) up to 2273 m^2^ g^−1^ and abundant micropores/mesopores in Fig. S1, which are the ideal nanostructures for electrocatalysis. To introduce nitrogen dopants and structural defects, the AC was annealed at 1000 °C for 2 h with a mixture of 2, 6-diaminopyidine/NaCl/ZnCl_2_, yielding a nitrogen-doped carbon catalyst with abundant defects, referred to as N-C_D_′. This material was then subjected to a flash Joule heating process for 1s at a heating rate of 800 °C s^−1^ to enhance its graphitization and structural properties (Fig. S2), resulting in the final catalyst, N-C_D_ (Fig. 1a). Control samples were prepared under similar conditions for comparative analysis, including a pure carbon catalyst (Pure C) without the addition of 2, 6-diaminopyidine/NaCl/ZnCl_2_, a carbon catalyst with defects but no nitrogen doping (Pure C_D_), and an N-doped carbon catalyst (N–C) prepared without NaCl/ZnCl_2_. Additionally, to evaluate the generalizability of the flash Joule heating process, various natural biomass-derived ACs, such as cork, bamboo, and poplar, were processed under identical conditions. The structural transformation of N-C_D_ compared to N-C_D_′ was evidenced by high-resolution transmission electron microscopy (HR-TEM) (Fig. 1b), which revealed an increased density of ordered lattice fringes, indicative of enhanced *sp*^2^-C domains content. Aberration-corrected transmission electron microscopy (ACTEM) (Fig. 1c) further confirmed this transformation, revealing the formation of many regular hexagon areas accompanied by an enhanced (002) Fast Fourier transform (FFT) pattern (Fig. 1d), indicative of improved graphitization [34]. In contrast, all control samples retained the amorphous carbon structure typical of biomass-derived materials, featuring abundant pores and short-range ordered lattice lines (Fig. S3). Energy-dispersive spectroscopy (EDS) elemental mapping demonstrated the uniform distribution of nitrogen dopants across the surface of N-C_D_ (Fig. S4), verifying the successful integration of heteroatoms.

**Fig. 2:**
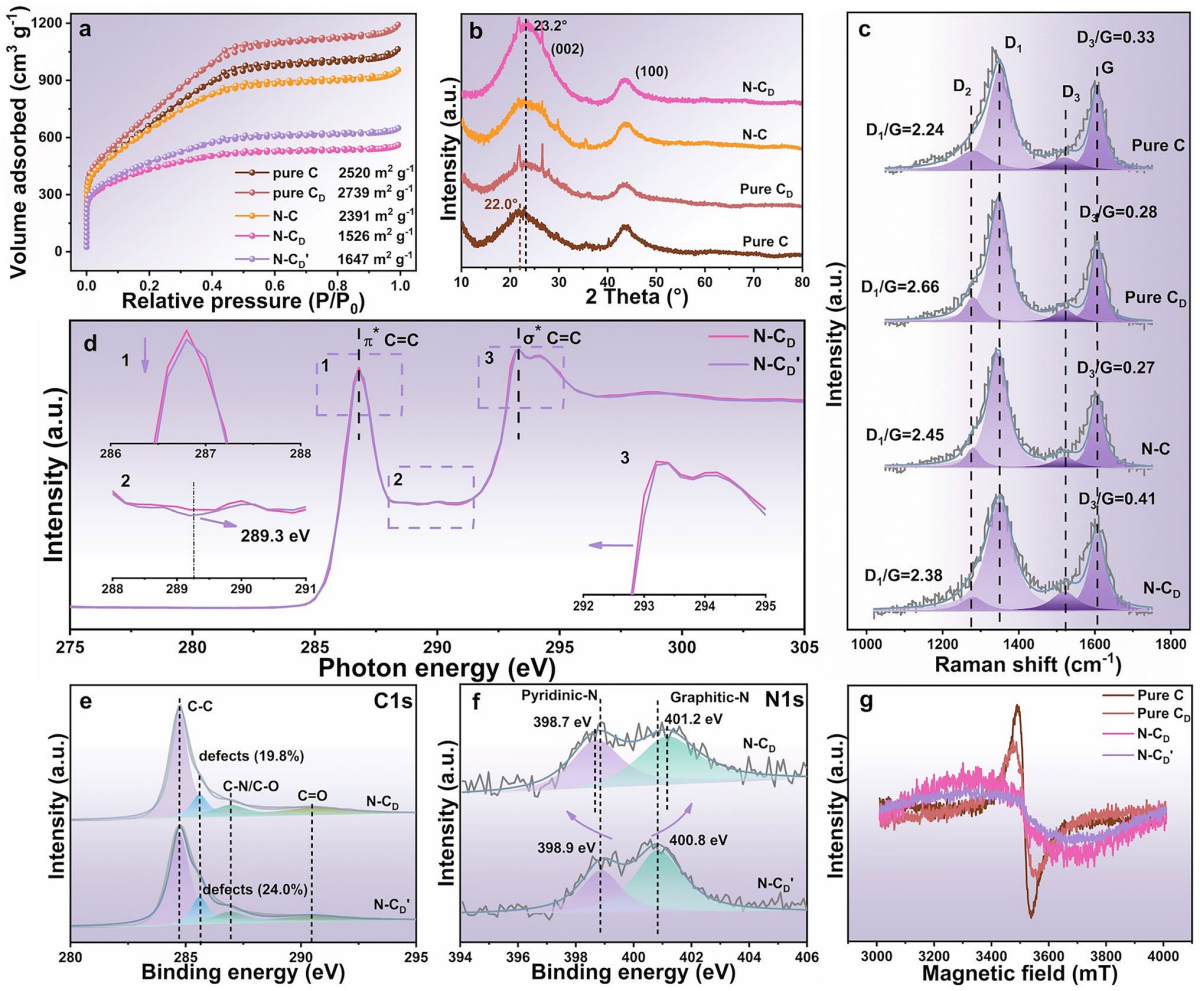
**a**–**c** N_2_ adsorption–desorption isotherms, XRD patterns and Raman spectra of Pure C, Pure C_D_, N–C and N-C_D_. **d**-**f** XANES spectrum of C K-edge, high-resolution XPS C 1*s* spectra and N 1*s* spectra of N-C_D_′ and N-C_D_. **g** EPR signals of different samples

The collapse of the porous structure and the reconstruction of carbon rings are well-documented phenomena observed following flash Joule heating treatment. In the case of N-C_D_, this process resulted in a reduction of its SSA to 1526 m^2^ g^−1^, as well as a decrease in pore content (Figs. 2a and S5). Despite this reduction, the SSA of N-C_D_ remains higher than that of most carbon-based catalysts, retaining its structural advantages. X-ray diffraction (XRD) analysis (Fig. 2b) revealed a pronounced increase in the intensity of the (002) peak for N-C_D_, along with a shift in the corresponding 2*θ* angle to 23.2° (versus 22° for the Pure C), indicating a higher degree of graphitization and improved crystallinity, which can be attributed to the reconstruction of *sp*^2^-C domains facilitated by the removal of nitrogen dopants during the flash Joule heating process[35]. Notably, the N–C and Pure C_D_ samples display lower (002) peak intensities, further underscoring the role of nitrogen doping in promoting *sp*^2^-C reconstruction during the heating process [36].

The Raman spectra of the samples (Fig. 2c) were deconvoluted into four characteristic bands: D_1_ (carbon defects), D_2_ (disorder graphitic lattice, A_1g_ symmetry), D_3_ (pentagons and N dopants), and G (graphitic lattice vibrations) [4, 14]. The N-C_D_ sample displayed a lower D_1_/G ratio (2.38) and a higher D_3_/G ratio (0.41), indicating a higher degree of graphitization and an increased density of geometric carbon defects/ nitrogen dopants, respectively. Normalized C K-edge X-ray absorption near-edge structure (XANES) spectra (Fig. 2d) provide further insights into the structural evolution. Compared to the N-C_D_′, the N-C_D_ sample exhibited a stronger π* peak (the electron transition of C 1*s* to unoccupied π* states) at 286.2 eV (region 1), representing the higher level of *sp*^2^-C configuration [14]. Simultaneously, a slight elevation in the peak at 288.3 eV (region 2), associated with *sp*^3^-C configurations, was also observed. While high-temperature annealing typically reduces *sp*^3^-C content by eliminating C-N and C-O bonds [4], the flash Joule heating process may retain residual N configurations that activate adjacent *sp*^2^-C atoms, creating a localized electronic environment resembling *sp*^3^-C structures. This hypothesis is further supported a slight negative-shift in the σ* peak at 293.4 eV (region 3) [14]. XPS analysis corroborates these findings, revealing a decrease in oxygen content (from 5.80 to 4.62 at%) and an increase in N content (from 1.84 to 2.35 at%) in N-C_D_ compared to N-C_D_′ (Table S1). In the high-resolution C 1*s* spectra (Fig. 2e), the defect peak content of N-C_D_ decreased from 24.0% to 19.8%, consistent with the observed reduction in *sp*^3^-C defects as indicated by Raman and XANES analyses.

The flash Joule heating process significantly altered the local electronic environments of nitrogen configurations, as revealed by shifts in binding energy observed in the high-resolution N 1*s* spectra (Fig. 2f). Specifically, the binding energy of pyridinic N decreased from 398.9 to 398.7 eV, while that of graphitic N increased from 400.8 to 401.2 eV, indicating a more graphitized nitrogen environment [37]. Similar N configuration was observed in the N–C catalyst (Fig. S6). Electron paramagnetic resonance (EPR) spectra provide additional insights into the electronic environment (Fig. 2g). The progressive weakening of the EPR signal in Pure C, Pure C_D_ and N-C_D_ showed an increasing graphitization degree, representing more *sp*^3^-C reconstruction. Both N-C_D_′ and N-C_D_ exhibited large linewidths, suggesting faster spin relaxation time caused by localized π electrons trapped at defects or vacancies, coupled with mobile electrons in extended aromatic domains [38]. Notably, the N-C_D_ showed wider and stronger EPR signal compared to N-C_D_′, indicating the synergistic effects of N configuration and localized *sp*^2^-C domain induced by the flash Joule heating treatment.

### Electrocatalytic Performance

**Fig. 3:**
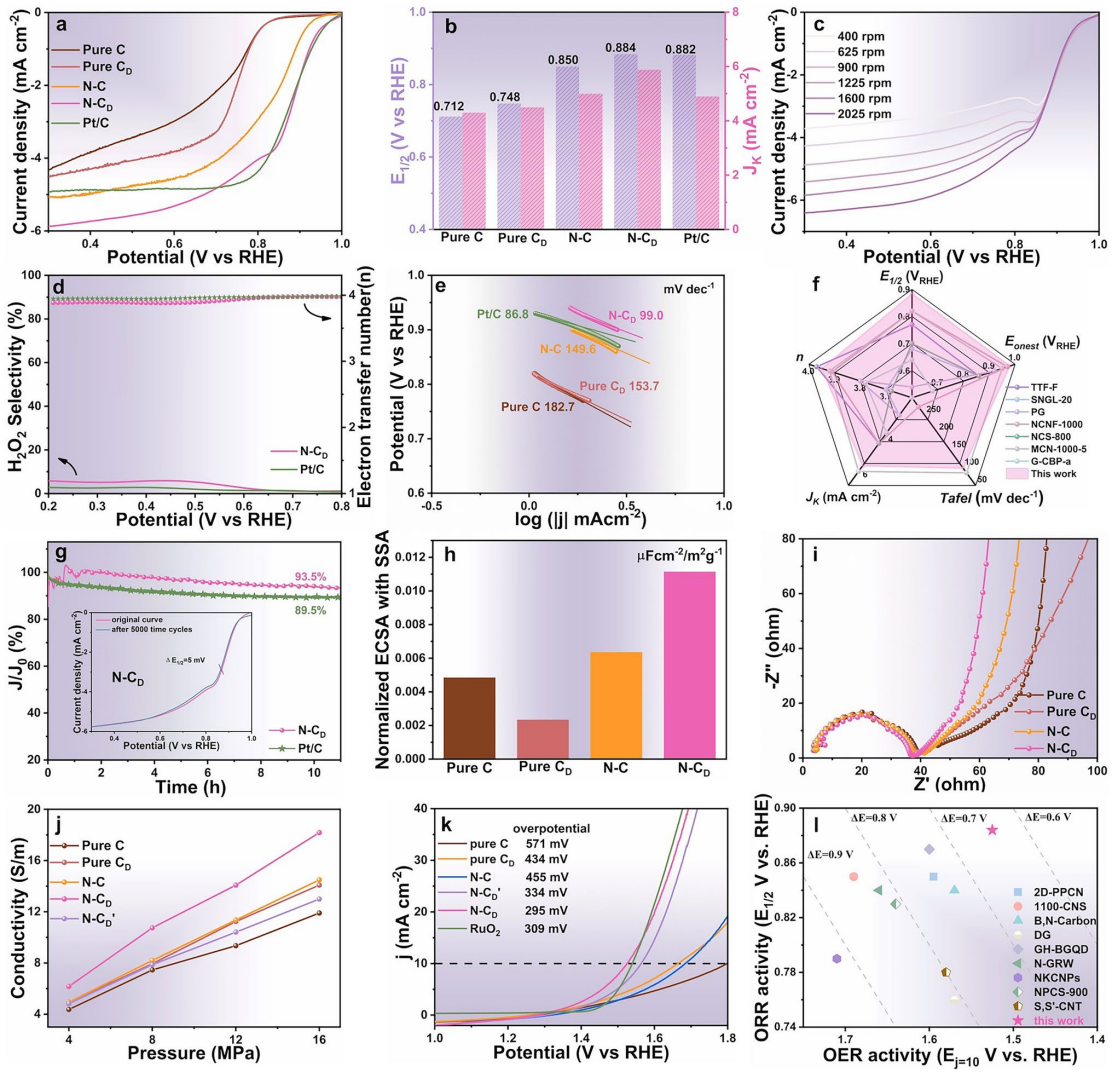
**a** LSV curves without iR compensation in O_2_-saturated 0.1 M KOH at the scan rate of 10 mV s^−1^ at 1600 rpm with RDE for carbon catalysts and 20% Pt/C. **b** *E*_1/2_ and *J*_k_ for different samples. **c** LSV curves of N-C_D_ at different rotation rates. **d** Electron transfer number (dotted line) and peroxide yield (H_2_O_2_%) (solid line) of N-C_D_ and 20% Pt/C measured with RRDE. **e** Tafel slopes based on LSV curves (**a**). **f** Radar plot for ORR performance comparison with the reported catalysts. **g** ORR durability measured for N-C_D_ by long-term chronoamperometric test in O_2_-saturated 0.1 M KOH (inset, continuous 5000 times CV scanning). **h** Normalized ECSAs values with SSAs for different samples. **i**, **j** EIS spectra and conductivity diagram under different pressure. **k** OER LSV curves in 1.0 M KOH for carbon catalysts and commercial RuO_2_ catalyst. **l** Comparison of catalytic performance with many reported ORR-OER bifunctional catalysts

The ORR electrocatalytic performance of N-C_D_ was measured using a rotating disk electrode (RDE) in a 0.1 M KOH electrolyte. The interference of residue Zn metal on the ORR catalytic performance was excluded through a poisoning experiment using KSCN (Fig. S7) [37]. As expected, N-C_D_ shows a higher ORR performance than that of N-C_D_′ (Fig. S8). The half-wave potential (*E*_1/2_) of N-C_D_ reached 0.884 *V*_RHE_, matching the performance of the commercial 20% Pt/C catalyst (0.882 *V*_RHE_) and surpassing those of control catalysts, including Pure C (0.712 *V*_RHE_), Pure C_D_ (0.748 *V*_RHE_) and N–C (0.850 *V*_RHE_) catalysts (Fig. 3a, b). Nevertheless, the N-C_D_ possesses higher current density of 5.88 mA cm^−2^ at 0.3 *V*_RHE_ than that of Pt/C (4.80 mA cm^−2^). The electron transfer number (n) for ORR, calculated from RDE data using K-L equation, was above 3.9 (Figs. 3c and S9), indicating a nearly complete 4-electron reduction pathway (Fig. 2d) [39]. This was further corroborated by RRDE measurements (Fig. S10), which showed a low H_2_O_2_ selectivity (< 5%) and an electron transfer number up to 3.90. Additionally, the Tafel slope of N-C_D_ (99.0 mV dec^−1^) outperform the control carbon catalysts and approach the Pt/C benchmark (86.8 mV dec^−1^), demonstrating the excellent ORR kinetics (Fig. 3e). We also annealed the N-C_D_′ at 800 °C in a tube furnace, and although it showed similar N content to the N-C_D_ catalyst, its ORR performance was inferior (Fig. S11 and Table S2). This further highlights the enhanced performance achieved by the Joule heating strategy.

To comprehensively evaluate the overall ORR performance, N-C_D_ was compared with previously reported carbon catalysts in terms of *E*_*1/2*_ (V_RHE_), onset potential (*E*_*onset*_, V_RHE_), kinetic current density (*J*_*K*_, mA cm^−2^), electron transfer number (n), and Tafel slope (mV dec^−1^), as summarized in a radar plot (Fig. 3f). The results clearly highlight the superior overall ORR performance of N-C_D_ compared to the state-of-the-art carbon-based catalysts.

The long-term catalytic stability of N-C_D_ was measured by chronoamperometry (*i-t*) and repeated CV cycling tests in Fig. 3g. After an 11-h test, the N-C_D_ retained 93.5% of its initial current density, outperforming Pt/C (89.5%). Additionally, the N-C_D_ exhibited only 5 mV (*E*_1/2_) reduction after 5000 CV cycles (Fig. 3g inset), further emphasizing its excellent catalytic stability. Consistent with the characteristics of metal-free carbon catalysts, the N-C_D_ shows excellent resistance to methanol poisoning in Fig. S12. The electrochemically active surface areas (ECSA) were evaluated using double-layer capacitance (*C*_dl_) method (Fig. S13) and normalized by BET specific surface area (Fig. 3h). The N-C_D_ demonstrated the highest normalized ECSA value, which was 1.7, 4.7, 2.3, and 1.9 times higher than those of N–C, Pure C_D_, Pure C and N-C_D_′, respectively. This result suggests the presence of abundant electrochemically active sites, attributed to the synergistic effect of *sp*^2^-C domain modulated by carbon defects and nitrogen dopants. The enhanced graphitization degree and increased N doping in N-C_D_ also contributed to decreased resistance and improved ion transport, as demonstrated by the electrochemical impedance spectra (EIS) in Fig. 3i. Additionally, the conductivity of N-C_D_, measured using a powder resistivity tester, was the highest among all samples (Fig. 3j), further corroborating the enhanced graphitization degree achieved through flash Joule heating.

For practical applications as a rechargeable air electrode in metal-air battery or fuel cell, a bifunctional catalyst capable of catalyzing both ORR and OER is highly desirable [3]. In OER performance tests, the N-C_D_ exhibited a significantly lower overpotential of 295 mV at 10 mA cm^−2^ (*E*_*j*_ = 10), which is substantially lower than the values of other control catalysts (571 mV for Pure C, 434 mV for Pure C_D_, 455 mV for N–C) and the commercial RuO_2_ catalyst (309 mV), disclosing a superior OER performance (Fig. 3k). Despite its excellent ORR performance, the relatively lower active site density in N-C_D_ leads to high Tafel slope (203 mV dec^−1^) compared to the RuO_2_ (116 mV dec^−1^), which is a general phenomenon for metal-free OER catalysts [40, 41] (Fig. S14). The bifunctional ORR-OER performance of N-C_D_ is further evaluated by potential difference ΔE (*E*_*j*=*10*_* − E*_*1/2*_). Remarkably, the ΔE of N-C_D_ is only 0.64 V much lower than those of the most reported metal-free bifunctional electrocatalysts (Fig. 3l and Table S3).

### Formation Mechanism of *sp*^2^-C Domains

**Fig. 4:**
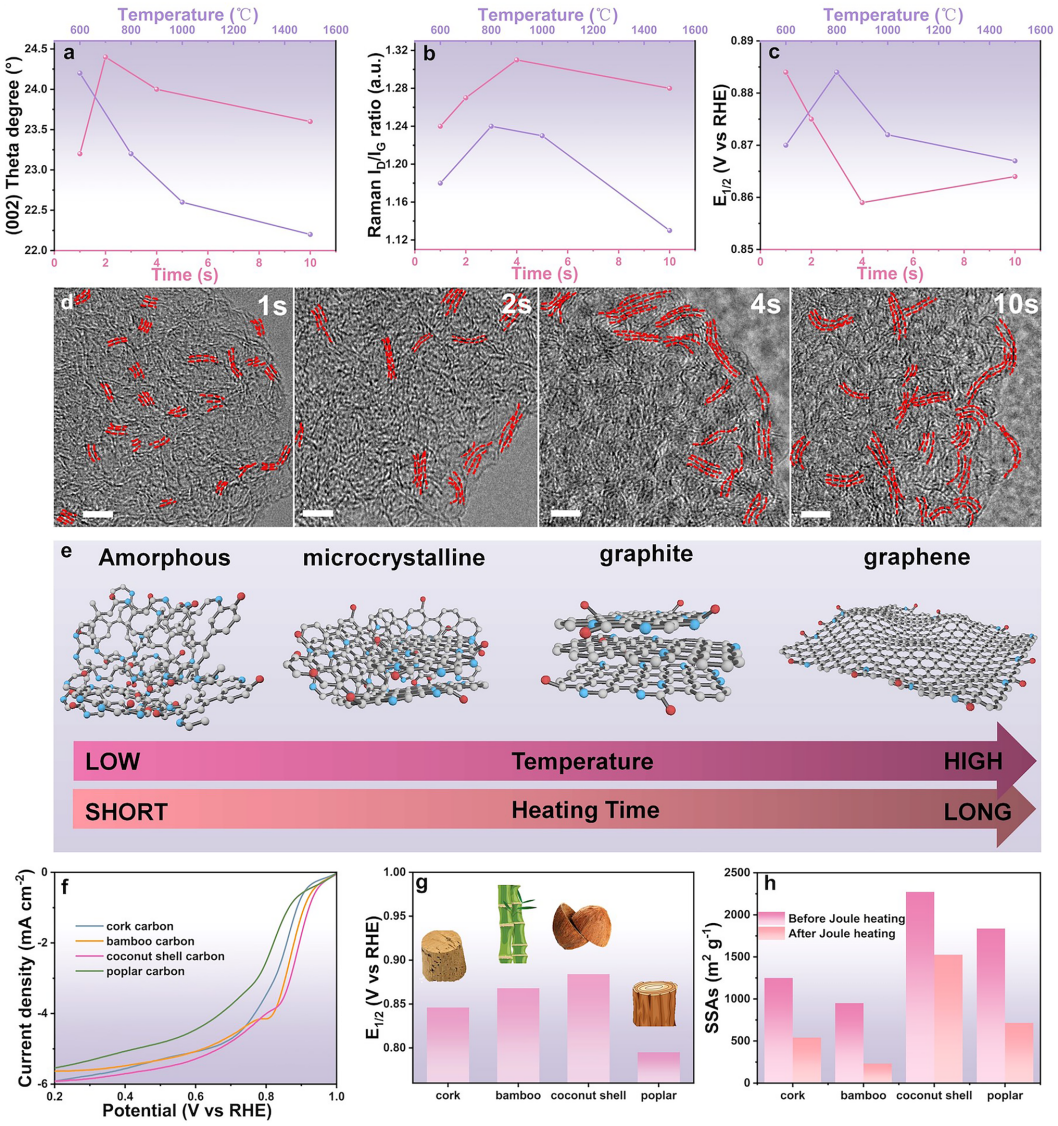
**a**–**c** Effect of Joule heating time and temperature on **a** 2*θ* degrees of (002) plane in XRD patterns, **b** *I*_D_/*I*_G_ ratios in Raman spectra, and **c** ORR performance. **d** HR-TEM images of N-C_D_ under different Joule heating time from 1 to 10 s. **e** Schematic diagram of carbon nanostructure with prolonging Joule heating time and increasing heating temperatures. **f**, **g** LSV curves and onset potentials for different natural biomass-based carbon catalysts in O_2_-saturated 0.1 M KOH electrolyte. **h** SSAs change before and after Joule heating at 800 °C for 1s

To further investigate the influence of *sp*^2^-C domains on catalytic performance, we examined the effects of two key parameters (temperature rate and duration time) for the formation of *sp*^2^-C domains structure [42]. With the increasing temperature rate (800–1500 °C/1s), the 2*θ* degrees of (002) plane decreased, indicating an increase in the interlayer spacing of the (002) plane. Simultaneously, the *I*_D_/*I*_G_ ratios gradually decrease, representing the reduction of active site density in Fig. 4a, b. Through balancing the *sp*^2^-C domains and active site density, the optimized flash Joule heating conditions (800 °C /1s for 1s) were identified, achieving the highest ORR performance (Fig. 4c). Prolonged heating durations (1 to 10 s) under 800 °C at a rate of 800 °C s^−1^ further promoted the formation of graphene-like structures (Fig. S15). During this process, the 2*θ* degrees of (002) plane and the *I*_D_/*I*_G_ ratios exhibited a volcanic trend, suggesting that the *sp*^2^-C domains firstly formed larger graphite structure and then exfoliated into graphene-like structure during a rapid cooling (Fig. 4d, e). Under the Joule heating, the amorphous carbon with relatively unstable structure like pentagon, heptagon C rings was decomposed and produced many active C atoms, and at rapid cooling progress, the active C atoms gather together to form stable *sp*^2^-C domains structure accompanied by a weak graphite exfoliation process [43]. With the increasing Joule heating temperature, the content of active C atoms increases, resulting into large-size *sp*^2^-C domains after rapid cooling progress [42]. The transformation was accompanied by a progressive decrease in oxygen and nitrogen content, and an increase in geometric and edge defects, contributing to the observed volcanic trend in I_D_/I_G_ ratios.

To demonstrate the broad applicability of *sp*^2^-C domain modulation, we extended the flash Joule heating approach to a variety of biomass-based activated carbons (ACs), including cork AC, bamboo AC, and poplar AC. In all cases, the Joule heating treatment significantly enhanced *sp*^2^-C domains (Fig. S16), leading to improved ORR performance, as reflected by higher current densities and more positive onset potentials across all tested samples (Fig. 4f, g). However, due to the amorphous and porous structure in ACs, the pore structure of these carbon catalysts collapsed during the Joule heating process, leading to a decrease in SSAs (Fig. 4h and S17).

### Mechanism Analysis of *sp*^2^-C Domains to Catalytic Performance

**Fig. 5:**
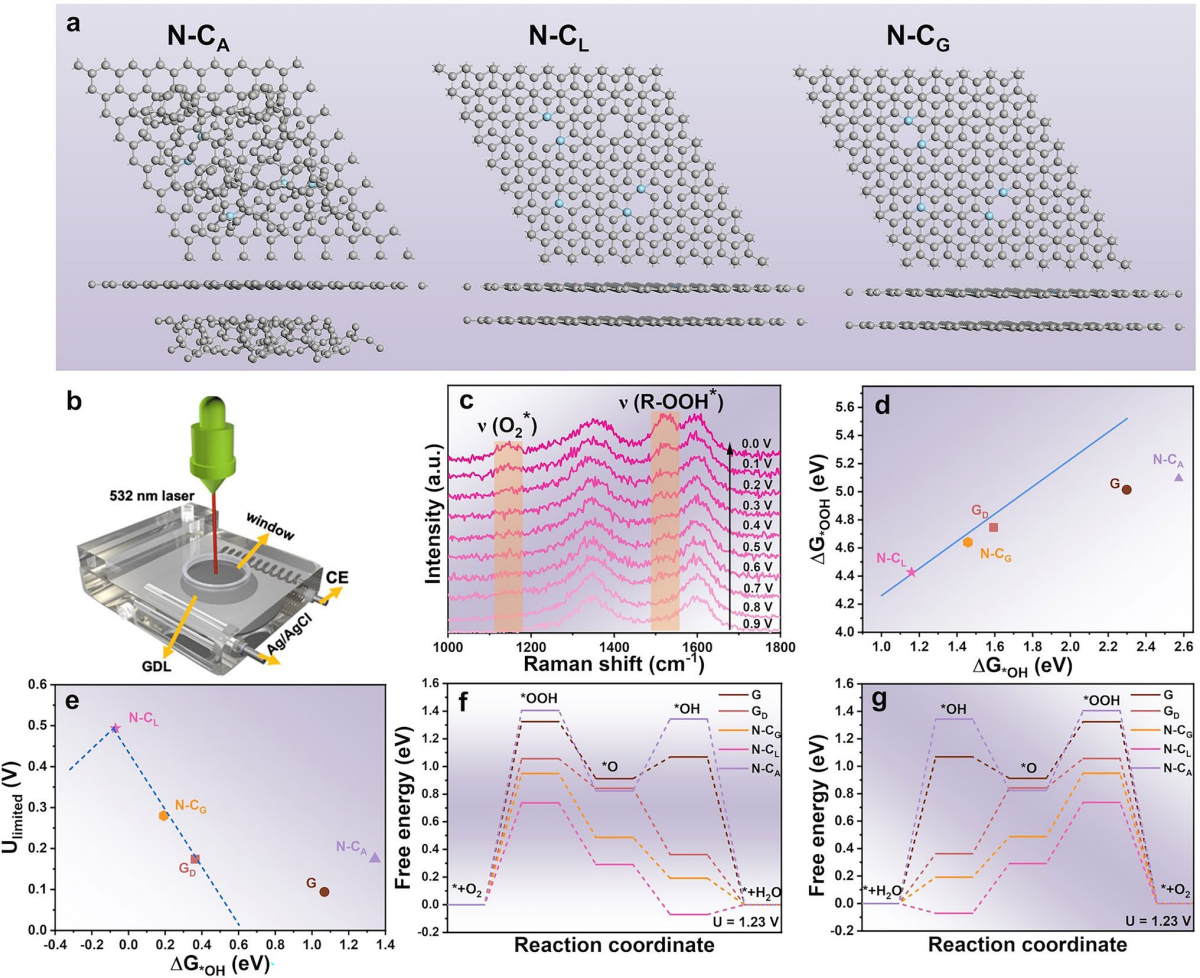
**a** DFT calculation models of N-C_A_, N-C_L_, N-C_G_. **b** Setup for measuring in-situ Raman spectra. **c** In-situ Raman spectroscopic study of the *OOH intermediate on N-C_D_ at various potentials vs. . RHE in 0.1 M KOH. **d** Scaling relationship between ΔG_*OOH_ and ΔG_*OH_. **e** ORR volcano plots of theoretical onset potential versus ΔG_*OH_. **f** Gibbs free energy of the ORR intermediates on different catalysts at 1.23 V. **g** Gibbs free energy of the OER intermediates on different catalysts at 1.23 V

DFT calculations were conducted to elucidate the influence of local enhanced *sp*^2^-C domains on the catalytic activity in N-C_D_. Based on the previous structure analysis, we designed two-layer carbon model comprising an N-doped graphene layer and an adjacent amorphous carbon layer, a locally enhanced *sp*^2^-C carbon layer or a graphene layer, corresponding models defined as N-C_A_, N-C_L_ and N-C_G_, respectively (Fig. 5a). Control models, including graphene (G), defective graphene (G_d_) and amorphous N-doped carbon (A_N-C), were also designed for comparison in Fig. S18. *In-situ* Raman spectroscopy provided insights into the interaction of reaction intermediates with active sites during the ORR (Fig. 5b). The signals at 1140 and 1520 cm^−1^ intensify with decreasing the potential from 0.8 to 0 V_RHE_, which can be ascribed to the O–O stretching vibration of the O_2_^−^ species and adsorbed *OOH, respectively (Fig. 5c) [44]. Deconvoluted Raman spectra (Fig. 2c) revealed a sharper D_3_ band (representing the amorphous carbon bonding with N atoms) under negative potentials, indicating an enhanced *OOH intermediate intensity on the active C atom sites modulated by N dopants and *sp*^2^-C domains. Moderate surface-oxygen interaction energy is essential for achieving high catalytic activity. The adsorption energies of *OOH and *OH need to be in an appropriate range during the 4e^−^ ORR process. According to the universal scaling relationship between ΔG_*OOH_ and ΔG_*OH_ (ΔG_*OOH_ = 0.97 × ΔG_*OH_ + 3.29, established in Fig. 5d), a typical volcano-type curve of the theoretical ORR onset potential (U_limited_) vs. ΔG_*OH_ of catalysts could be plotted in Fig. 5e (dotted line) [26].

The N-C_L_, N-C_G_ and G_d_ simultaneously possess the suitable ΔG_*OOH_ and ΔG_*OH_, indicating their potential for high catalytic activity. Among these, the N-C_L_ catalyst was positioned at the peak of the volcano plot, indicating its superior catalytic activity, with the enhanced *OOH adsorption energy values optimized for efficient ORR kinetics [45]. The free energy of oxygen intermediates on catalyst surface was calculated and plotted at different voltages (*U* = 1.23 V and *U* = 0 V) (Figs. 5f and S19, S20). At (U = 0) V, the free energy decrease for N-C_L_ was 0.49 eV, substantially greater than the 0.28 eV observed for N-C_G_. When a voltage of 1.23 V is applied (*U* = 1.23 V), the potential-determining step (PDS) was identified as the *OOH adsorption (Fig. 5f). When the N sites combinate with local enhanced *sp*^2^-C domains, the adsorption barrier for *OOH (the PDS of the ORR at the catalysts) was significantly reduced, leading to an increase in the onset potential of ORR [14]. Specifically, the *OOH energy barriers for G, G_d_, N-C_G_, and N-C_L_ were 1.32, 1.05, 0.95, and 0.73 eV, respectively. Among these, N-C_L_ demonstrated the lowest energy barrier, attributed to the coupling effect between N doping and locally enhanced *sp*^2^-C domains.

Charge density difference mapping (Fig. S21) further revealed significant charge redistribution around *sp*^2^-C domains and N-doped sites in N-C_A_, N-C_L_, and N-C_G_, with evident charge accumulation and depletion enhancing O_2_ adsorption and activation. The optimized adsorption configurations of ORR intermediates (*, *OOH, *O, and *OH) on N-C_L_ (Fig. S22) confirmed its superior interaction with intermediates, boosting ORR catalytic activity. In OER reaction pathway (Fig. 5g), the free energy changes during each intermediate step were calculated. The N-C_L_ exhibited the lowest energy barriers throughout the OER process, particularly during the critical *O to *OOH transition, demonstrating a superior OER performance [4, 26]. The DFT calculations unravel that the synergy between N doping and locally enhanced *sp*^2^-C domains optimizes the electronic structure of active C sites, thereby enhancing catalytic performance in ORR and OER, which is consistent with our experimental results.

There are two kinds of N configurations (pyridinic N and graphitic N) in N-C_D_ sample, and therefore, we designed two kinds of model modulated by different size *sp*^2^-C domains in Fig. S23. As the size of *sp*^2^-C domain increases, the delocalization ability of its conjugated π-electron system enhances (evidenced by the increase in the density of states in DOS analysis in Fig. S24) [46]. This extended conjugated structure significantly modulates the electron cloud distribution of nearby N-doped sites through *π* interactions[47]. When pyridinic N is modulated by a large-size *sp*^2^-C domain, its lone pair electrons partially delocalize into the adjacent *sp*^2^-C backbone, resulting in a decrease in the charge density of pyridinic N site [48]. This charge redistribution optimizes the adsorption energy of O_2_ on the pyridinic N: the moderate electron-deficient nature promotes O_2_ adsorption while weakening the excessive binding of the *OOH intermediate, thereby enhancing ORR kinetics in Fig. S25.

### Application in Zn‐Air Battery

**Fig. 6:**
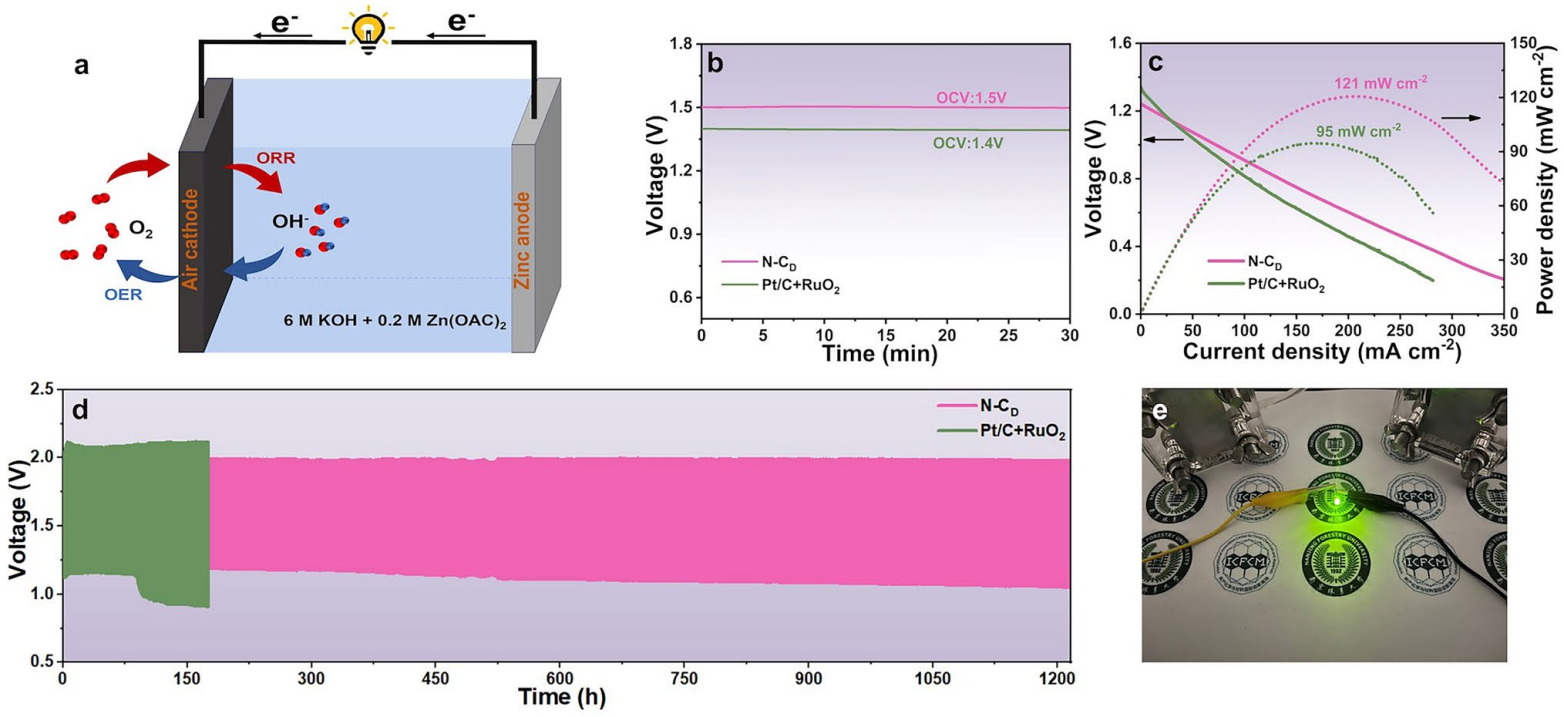
**a** Schematic configuration of the assembled ZAB. **b** Open-circuit voltages for ZABs using air cathodes of N-C_D_ or Pt/C + RuO_2_. **c** Discharge polarization curves and corresponding power density plots. **d** Discharge–charge cycling curves for the ZABs at 5 mA cm^−2^. **e** Photograph of a green LED powered by two N-C_D_ assembled ZABs

To probe the practical application of N-C_D_, a liquid Zn–air battery (ZAB) with N-C_D_ air cathode was assembled in a 6.0 M KOH electrolyte containing 0.2 M Zn(OAc)_2_ (Figs. 6a and S26) [49]. The battery achieved an open-circuit voltage of 1.5 V and a peak power density of 121 mW cm^−2^, significantly outperforming the commercial 20% Pt/C + RuO_2_ bifunctional catalyst, which exhibited an open-circuit voltage of 1.4 V and a peak power density of 95 mW cm^−2^ (Fig. 6b, c). Long-term stability testing was performed using galvanostatic charge and discharge cycling at a current density of 5 mA cm^−2^ with alternating 10 min charge and discharge cycles. After 80 h, the potential gap for the ZAB using Pt/C + RuO_2_ catalyst increases by 0.18 V. In stark contrast, the ZAB with the N-C_D_ catalyst exhibited excellent catalytic stability, with no noticeable degradation even after 1200 h of continuous operation (Fig. 6d). This demonstrates the exceptional catalytic stability of N-C_D_ during repeated ORR and OER cycles. Furthermore, the long-term stability of the N-C_D_-based ZAB surpasses that of previously reported bifunctional oxygen catalysts (Table S4). After long-term stability test, two N-C_D_ assembled ZABs successfully illuminated a green light-emitting diode, further underscoring the practical potential of this catalyst for sustainable energy applications (Fig. 6e).

## Conclusion

In summary, we demonstrate a significant advancement in oxygen electrocatalysis by enhancing *sp*^2^-C domains in N-doped biomass-derived carbon materials through a rapid flash Joule heating process (just 1s). This novel method effectively elevates the edge defect density and degree of graphitization within the carbon structure. The increased defect density optimizes the electronic structure of N configurations, creating highly active catalytic sites, while the enhanced graphitization facilitates rapid electron transfer, synergistically boosting catalytic efficiency. Consequently, the *sp*^2^-C enhanced N-doped carbon catalyst exhibited exceptional bifunctional performance. For the ORR, the optimized catalyst achieved a half-wave potential of 0.884 *V*_RHE_, equivalent to commercial 20% Pt/C, with a superior kinetic current density of 5.88 mA cm^−2^. For the OER, the catalyst displayed an overpotential of 295 mV at 10 mA cm^−2^, further underscoring its bifunctional capabilities. DFT calculations provided mechanistic insights, revealing that the enhanced catalytic performance is driven by axial regulation of electronic states at active carbon sites by local *sp*^2^-C domains. The practical applicability of this catalyst was validated in a Zn-air battery, which exhibited exceptional charge–discharge stability, maintaining performance for over 1200 h without detectable degradation, representing one of the most durable bifunctional oxygen electrocatalysts reported to date. This work elucidates the critical role and formatting mechanism of *sp*^2^-C domains in natural biomass-derived carbon materials processed via Joule heating process. Importantly, it establishes a universal and scalable strategy for modulating biomass-based carbon electrocatalysts, paving the way for their application in advanced energy storage and conversion systems.

## Supplementary Information

Below is the link to the electronic supplementary material.

Supplementary file1 (DOCX 32247 KB)
