# Supplementary material for: Joule Heating-Driven sp2-C Domains Modulation in Biomass Carbon for High-Performance Bifunctional Oxygen Electrocatalysis
Source: Nanomicro Lett. 2025 Apr 18;17:221. doi: 10.1007/s40820-025-01725-0 (PMC12006640; doi:10.1007/s40820-025-01725-0)
Supplement: Supplementary file 1 — Supplementary file1 (DOCX 32247 KB) [file 40820_2025_1725_MOESM1_ESM.docx]

Supporting Information for

**Joule Heating-Driven *sp^2^*-C Domains Modulation in Biomass Carbon for High-performance Bifunctional Oxygen Electrocatalysis**

Jiawei He^1#^, Yuying Zhao^3#^, Yang Li^4^, Qixin Yuan^1^, Yuhan Wu^1^, Kui Wang^3^, Kang Sun^3^, Jingjie Wu^4^, Jianchun, Jiang^3^, Baohua Zhang^5^, Liang Wang^2^*, Mengmeng Fan^1,3^*

^1^ Jiangsu Co-Innovation Center of Efficient Processing and Utilization of Forest Resources, International Innovation Center for Forest Chemicals and Materials, College of Chemical Engineering, Nanjing Forestry University, Nanjing 210037, P. R. China

^2^ Institute of Nanochemistry and Nanobiology, School of Environmental and Chemical Engineering, Shanghai University, Shanghai 200444, P. R. China

^3^ Key Lab of Biomass Energy and Material, Jiangsu Province; Jiangsu Co-Innovation Center of Efficient Processing and Utilization of Forest Resources, Institute of Chemical Industry of Forest Products, Chinese Academy of Forestry, Nanjing 210042, P. R. China

^4^ Department of Chemical and Environmental Engineering, University of Cincinnati, Cincinnati, OH 45221, USA

^5^ Department of Chemical Engineering, School of Environmental and Chemical Engineering, Shanghai University, Shanghai 200444, P. R. China

^#^Jiawei He and Yuying Zhao contributed equally to this work.

*Corresponding authors. E-mail: [wangl@shu.edu.cn](mailto:wangl@shu.edu.cn) (Liang Wang); [fanmengmeng370@163.com](mailto:fanmengmeng370@163.com) (Mengmeng Fan)

**Supplementary Figures and Tables**


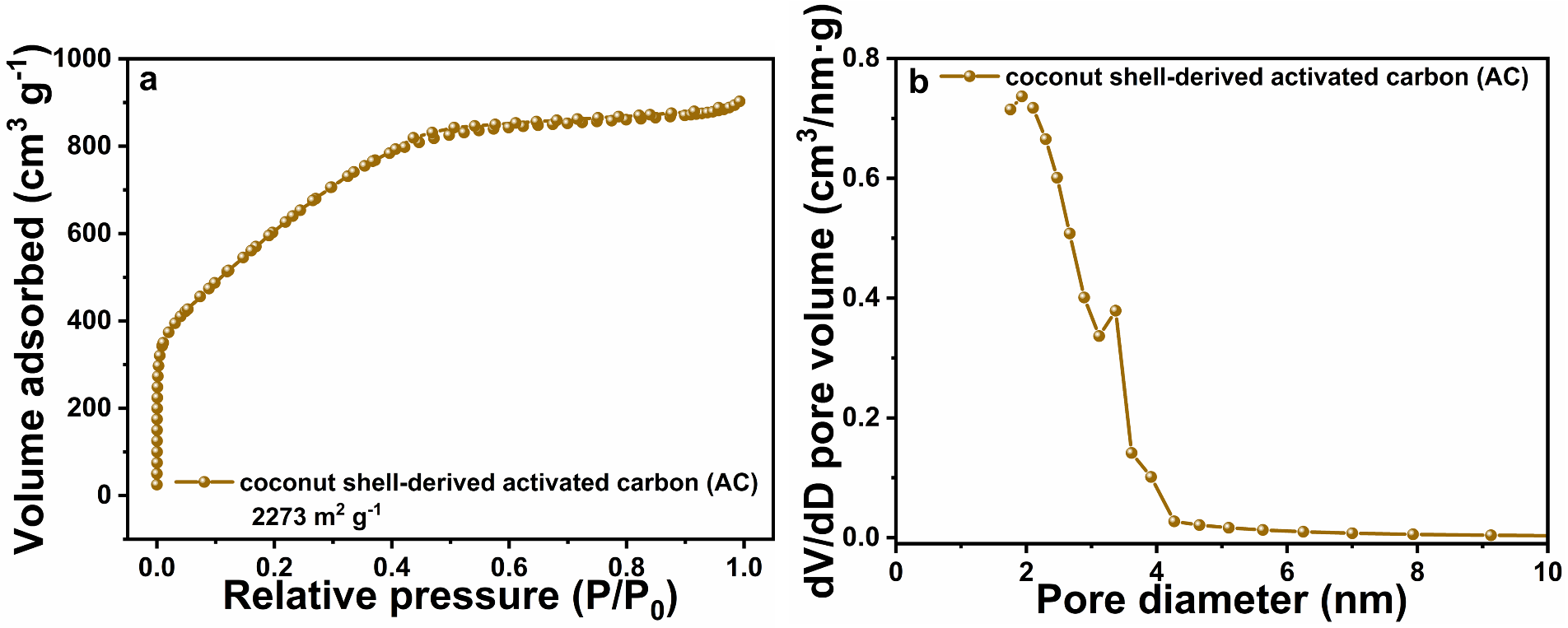


**Fig. S1** N_2_ adsorption–desorption isothermal curves (**a**) and pore size distribution (**b**) of coconut shell-derived activated carbon (AC)

**
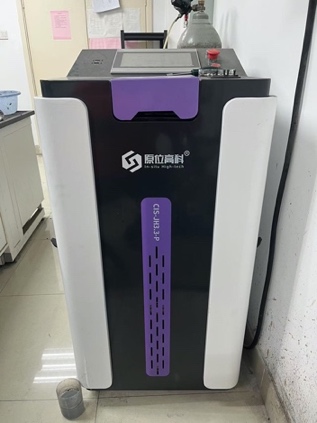
**
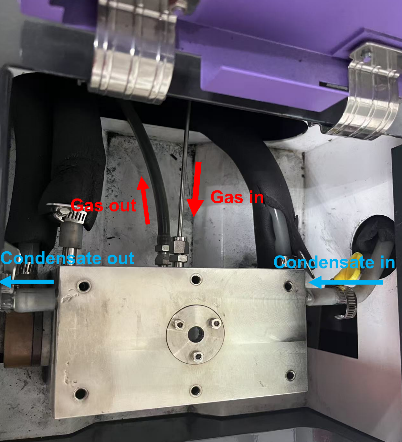

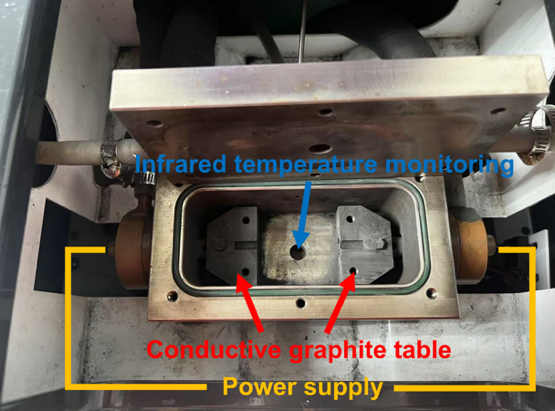




**Fig. S2** Joule heating equipment and the heating curves for N-C_D_ sample

**
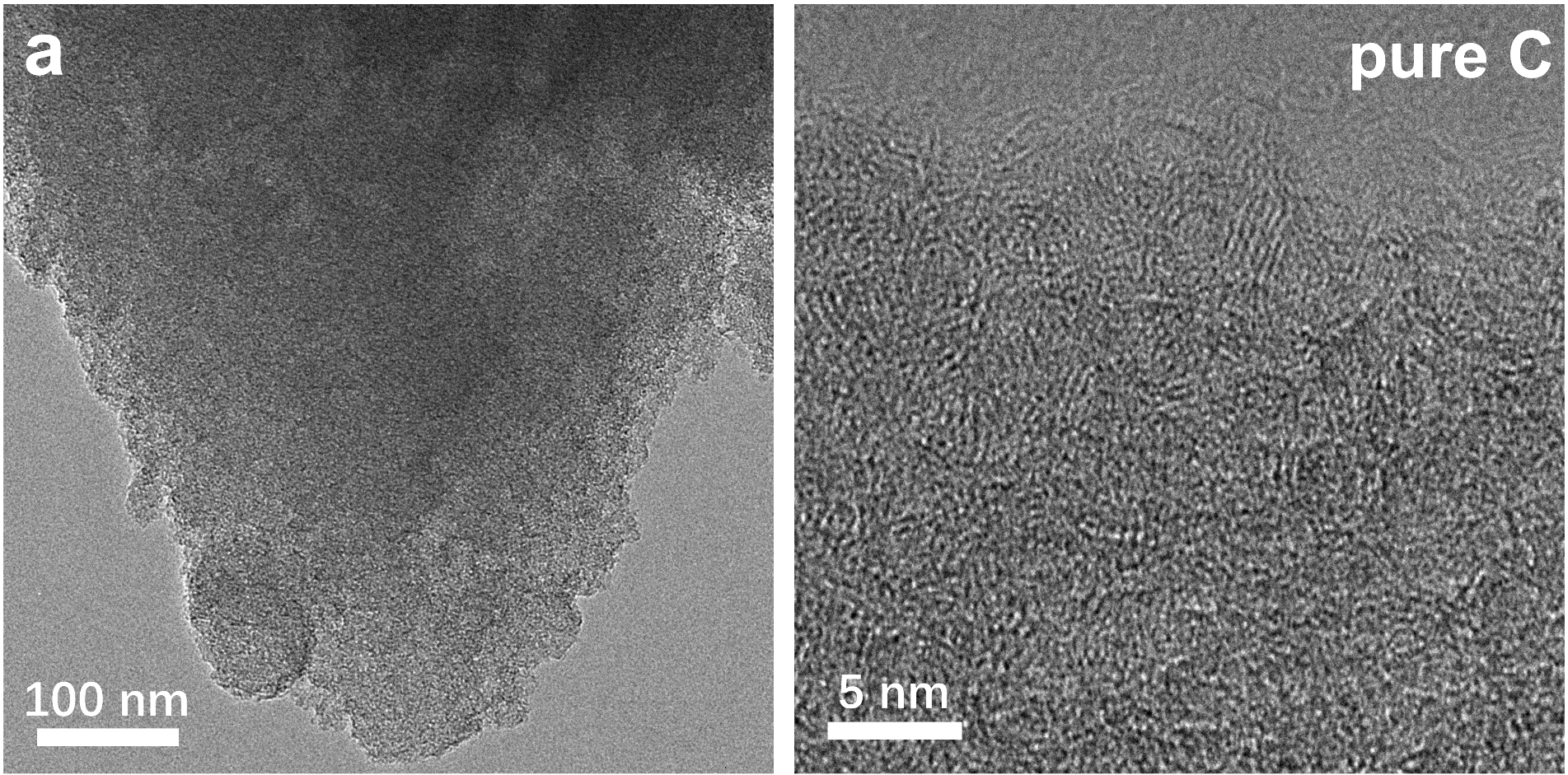

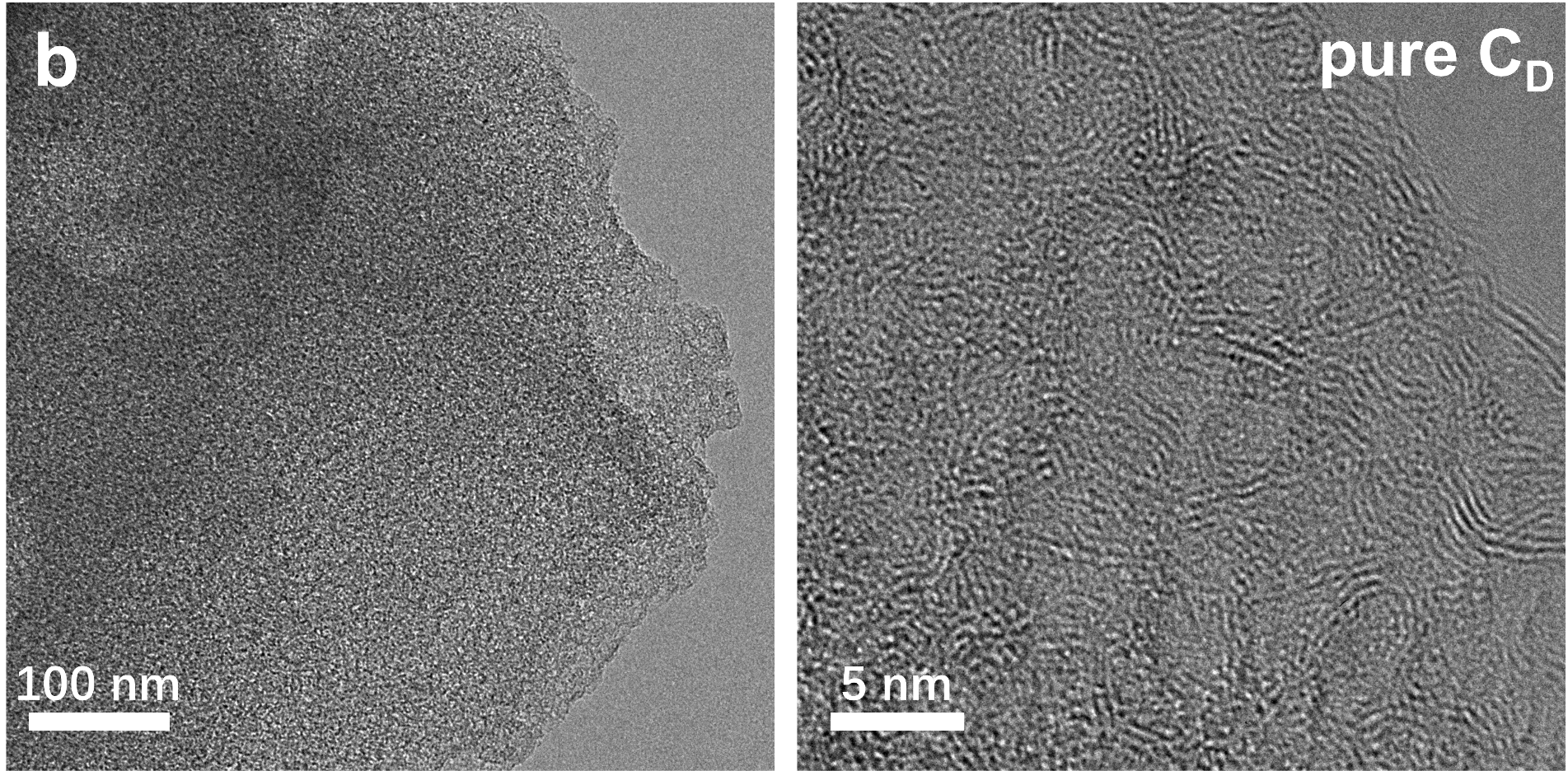

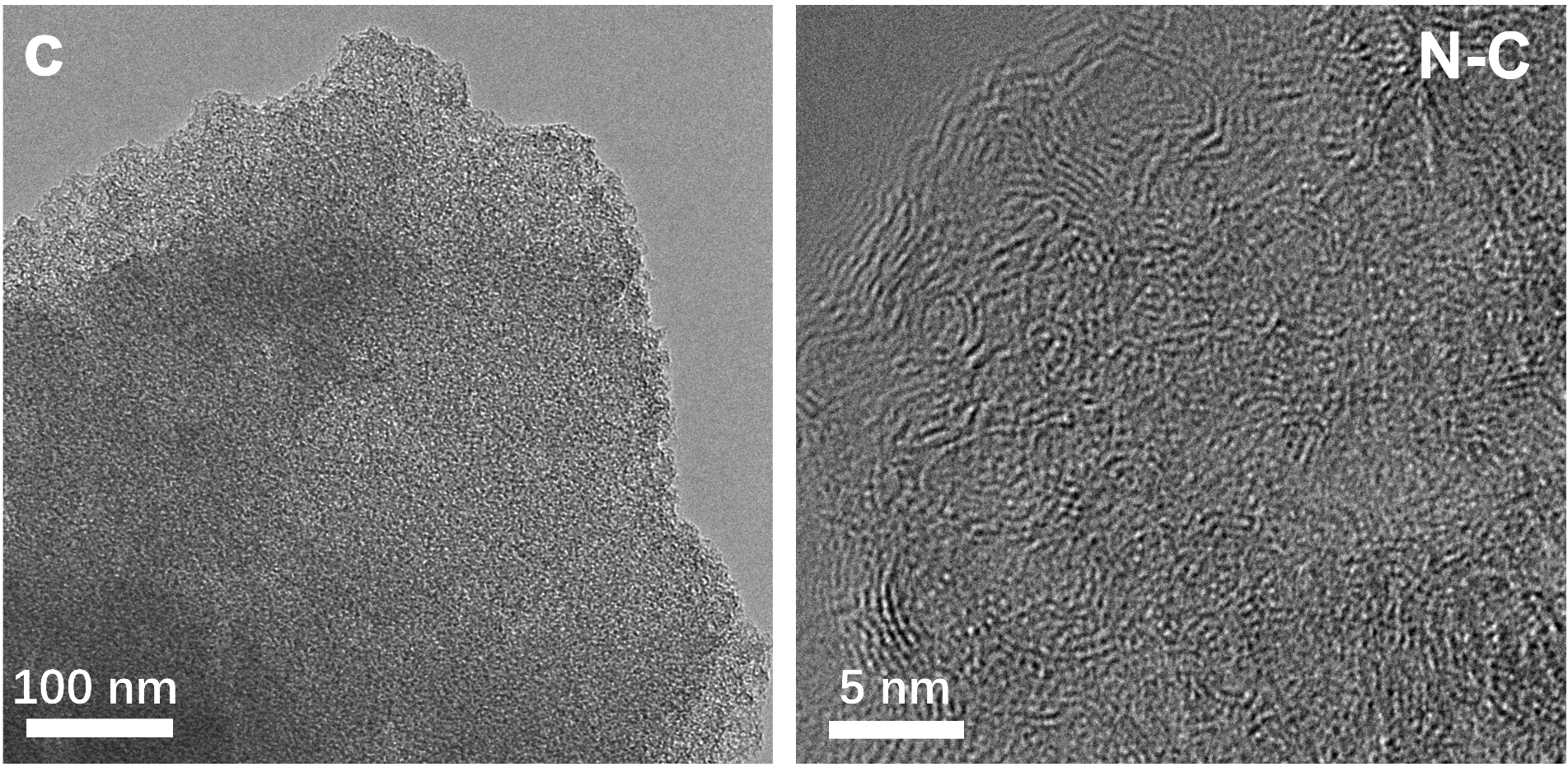

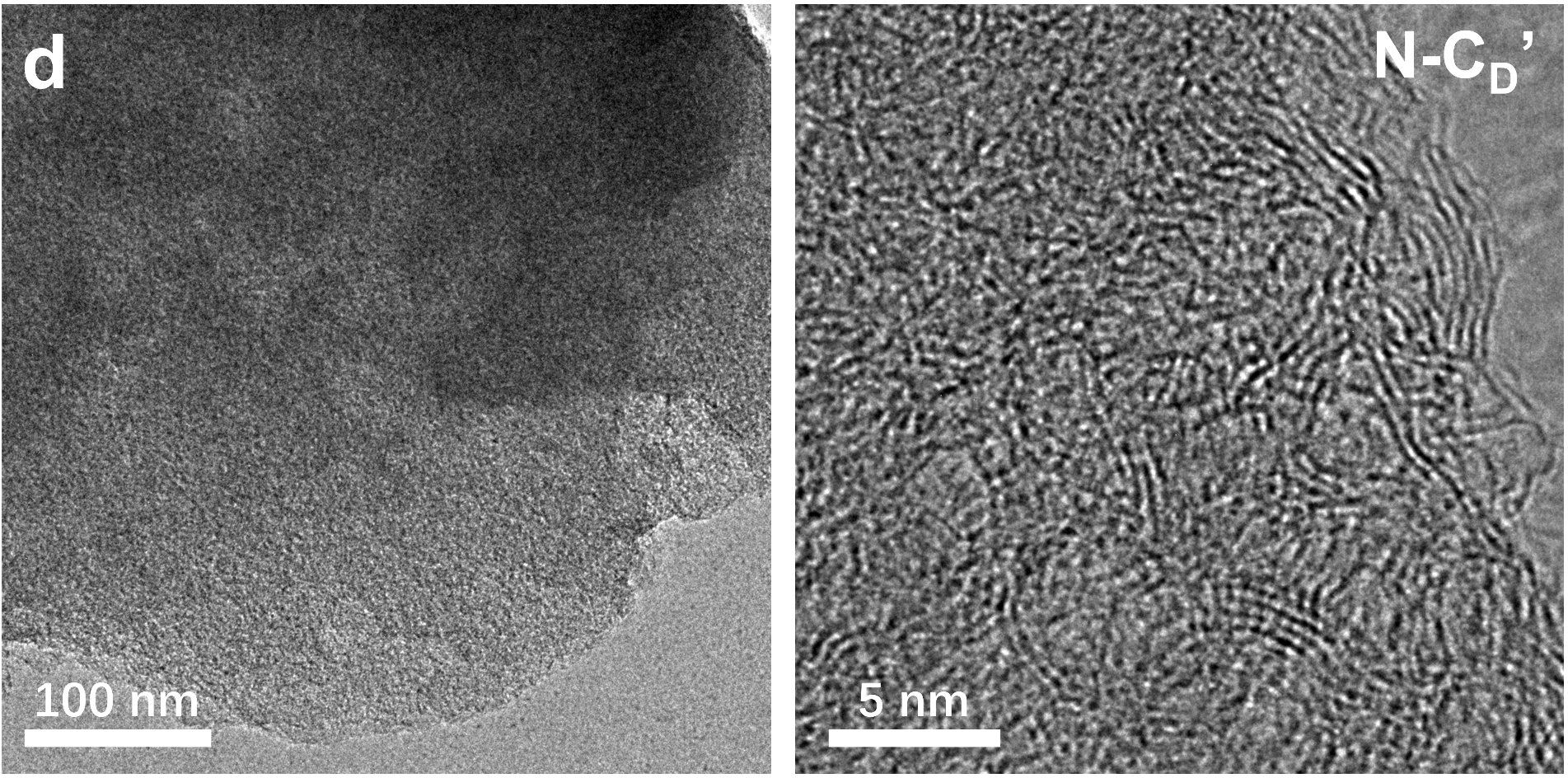
**

**Fig. S3** HR-TEM images of pure C (**a**), pure C_D_ (**b**), N-C (**c**) and N-C_D_’ (**d**)

**
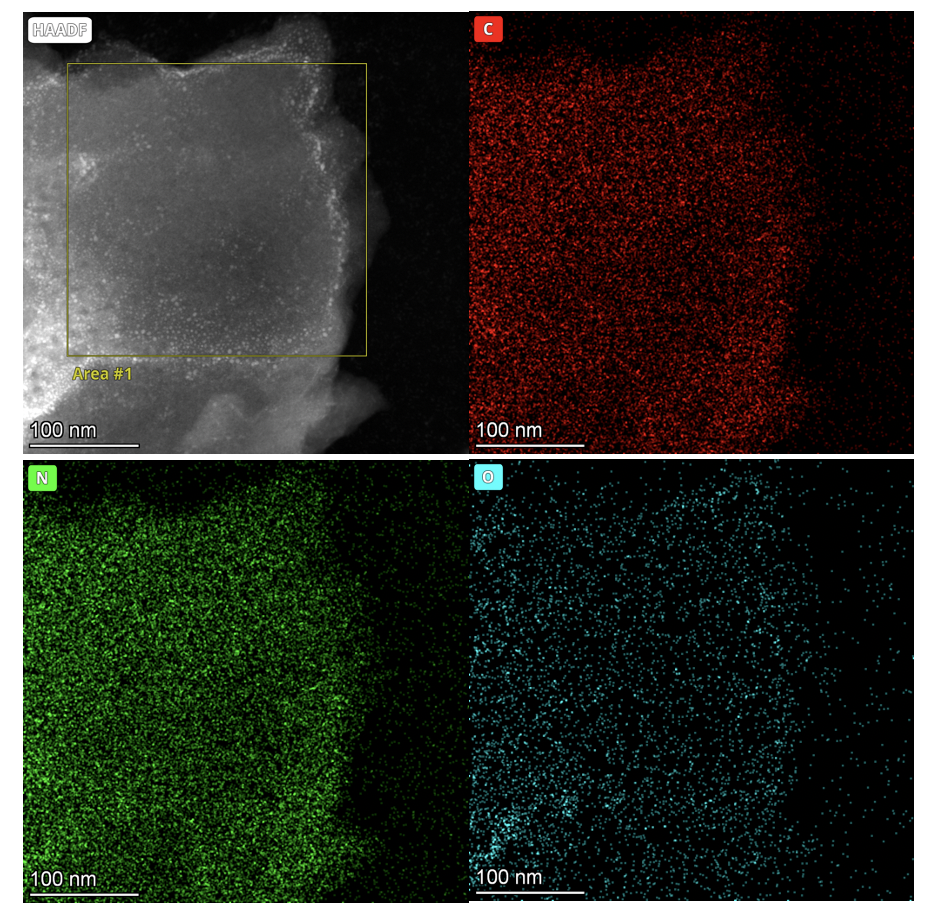
**

**Fig. S4** High-angle annular dark-field scanning transmission electron microscopy (HAADF-STEM) image and the corresponding EDS mapping images of N-C_D_





**Fig. S5** Pore size distribution of different samples





**Fig. S6** High-resolution N 1s spectra of N-C





**Fig. S7** LSV curve of N-C_D_ before and after adding 10 mM KSCN into O_2_-saturated 0.1 M KOH solution





**Fig. S8** LSV curves without iR compensation in O_2_ saturated 0.1 m KOH at the scan rate of 10 mV s^−1^ at 1600 rpm with RDE for N-C_D_ and N-C_D_’


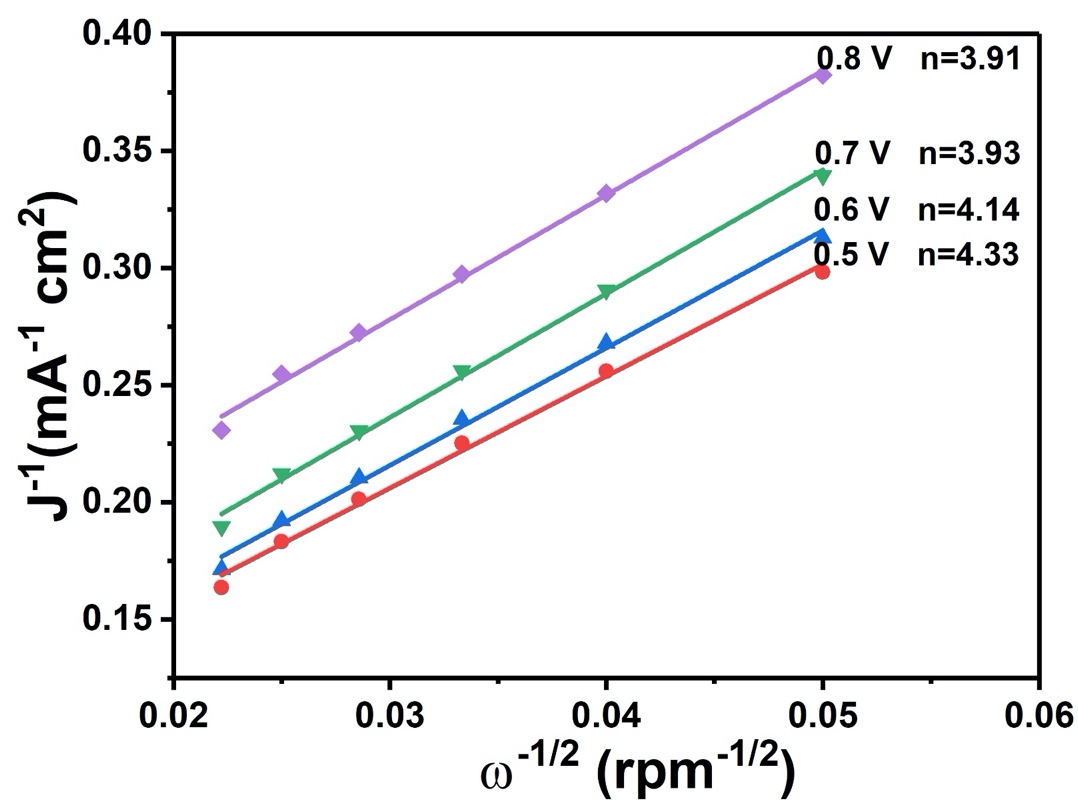


**Fig. S9** K-L plots and electron transfer numbers for N-C_D_





**Fig. S10** LSV curve (solid line) of N-C_D_ measured using a Rotating Ring-Disk Electrodes (RRDE) and the corresponding H_2_O_2_ current (dashed line) on the ring electrode were obtained in O_2_ saturated 0.1 M KOH at the speed of 1600 rpm with a fixed potential of 1.2 V vs. RHE


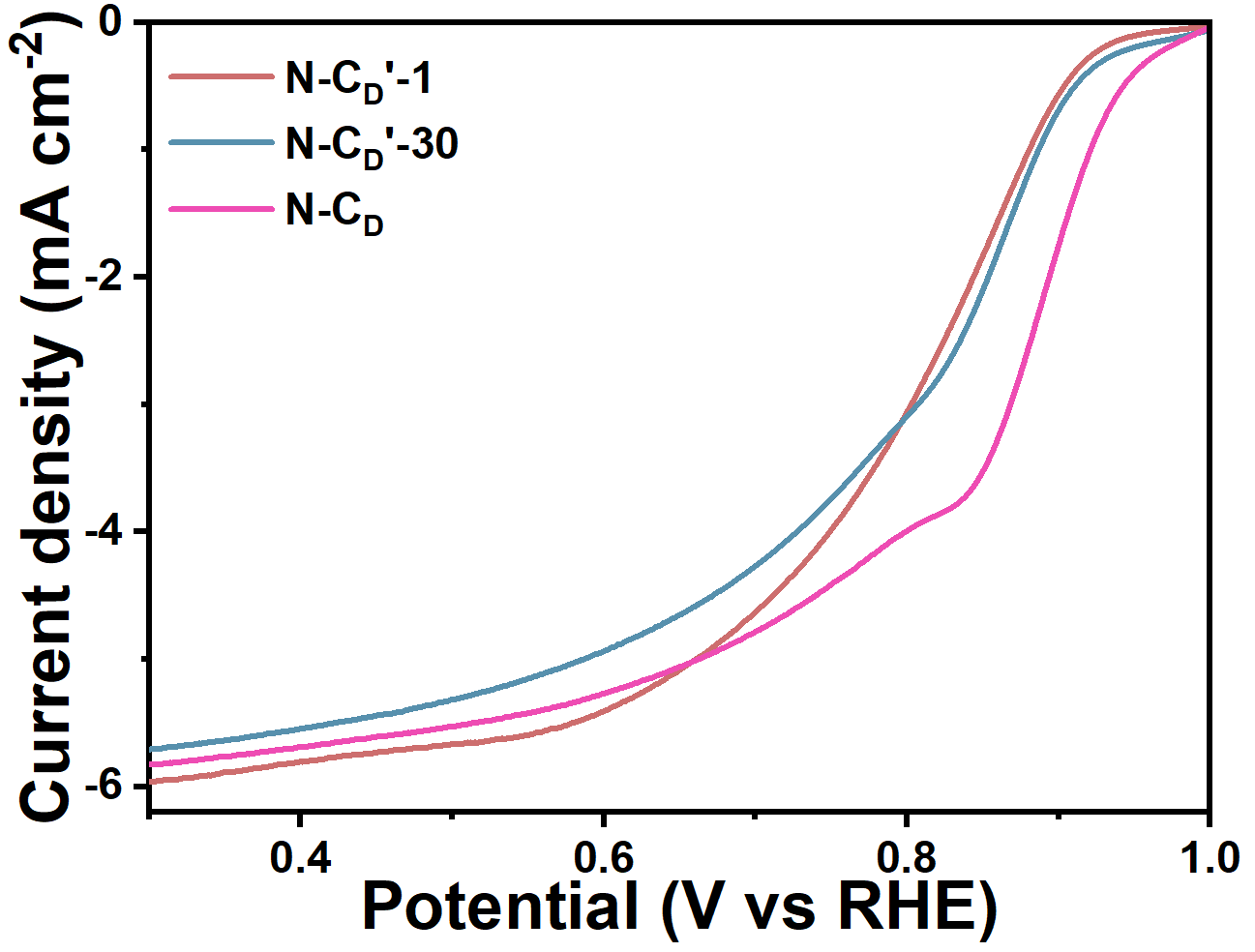


**Fig. S11** LSV curves of catalysts annealed in secondary tube furnaces





**Fig. S12** The anti-methanol toxicity test of N-C_D_ and Pt/C 20% adding 2 mL methanol at 30min in 0.1 M KOH


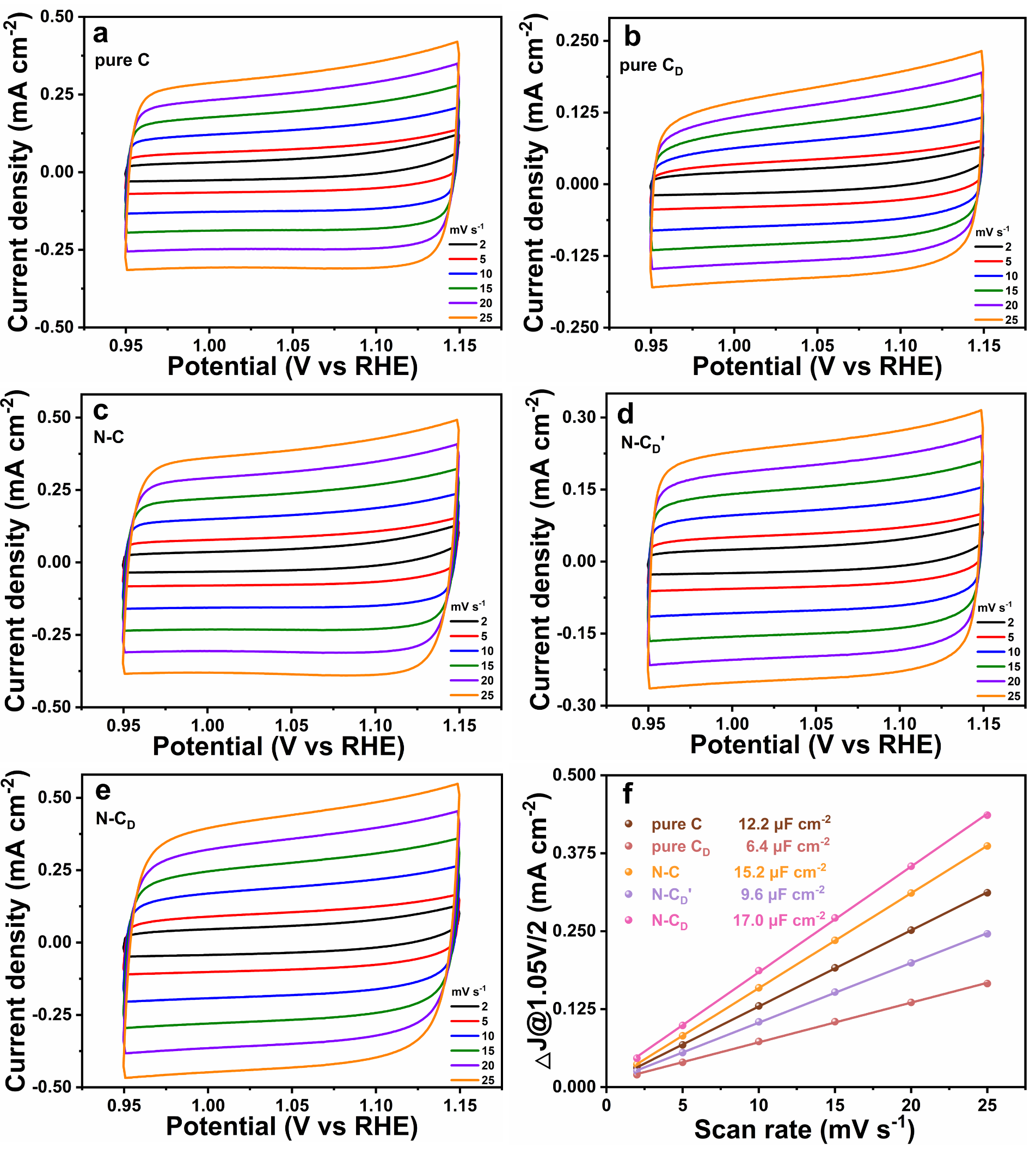


**Fig. S13** (**a-e**) CV curves obtained in a potential window of 0.95-1.15 V vs RHE at different scan rates in Ar saturated 0.1 M KOH for pure C (**a**), pure C_D_ (**b**), N-C (**c**), N-C_D_’ (**d**) and N-CD (**e**). (**f**) The charging current density plots with different scan rates for the samples


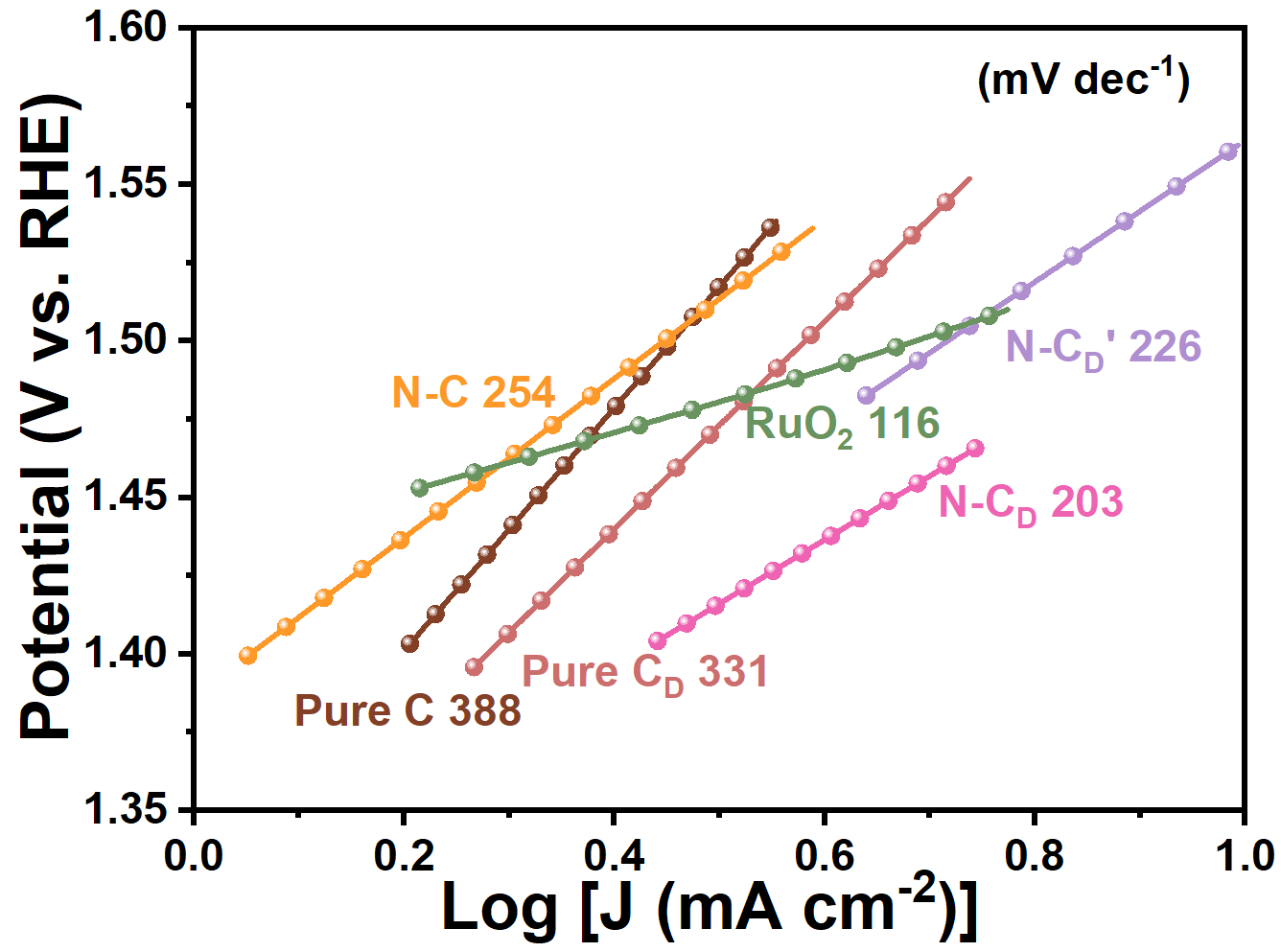


**Fig. S14** OER Tafel plots of the catalysts

**
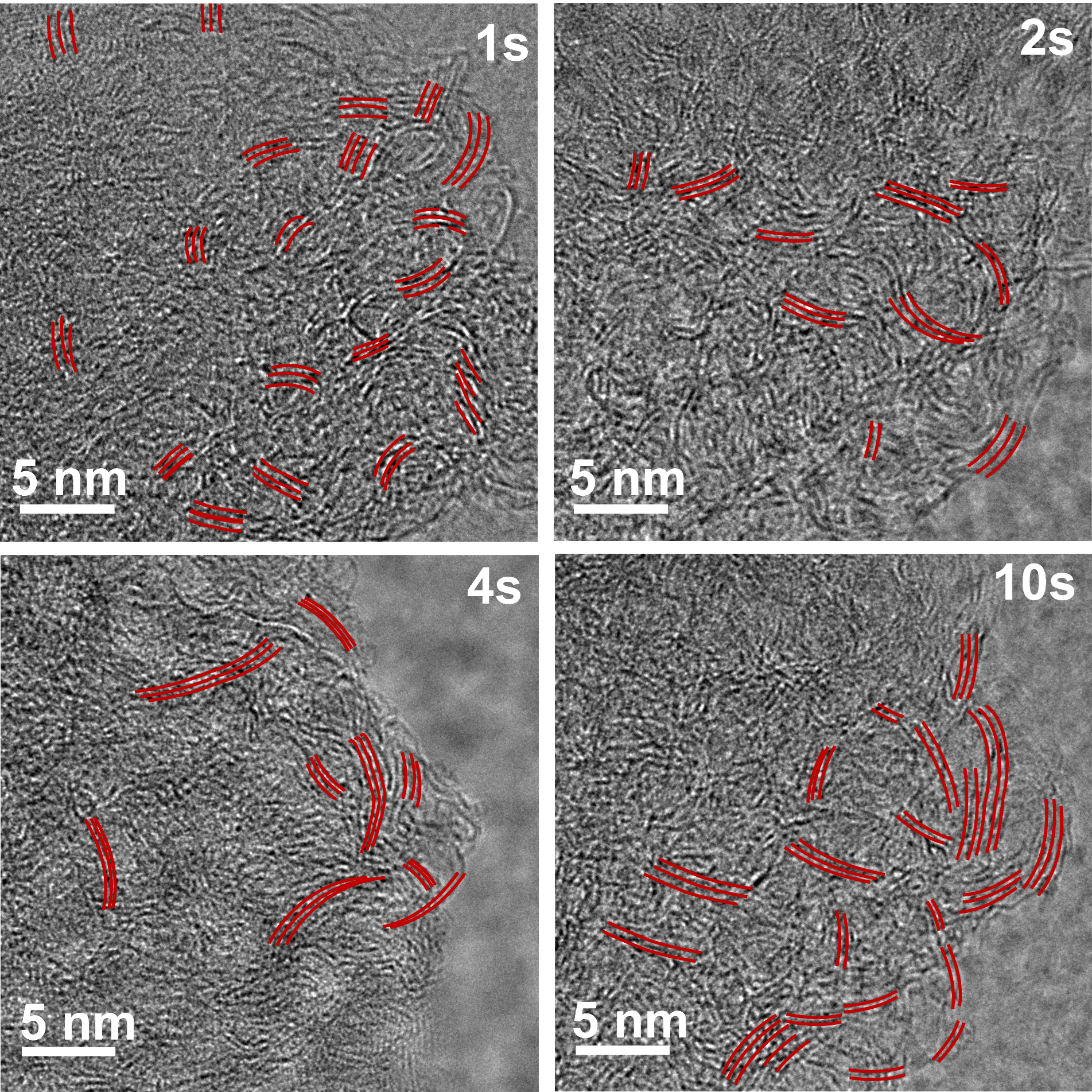
**

**Fig. S15** HR-TEM images of N-C_D_ under different joule heating time from 1 s to 10 s

**
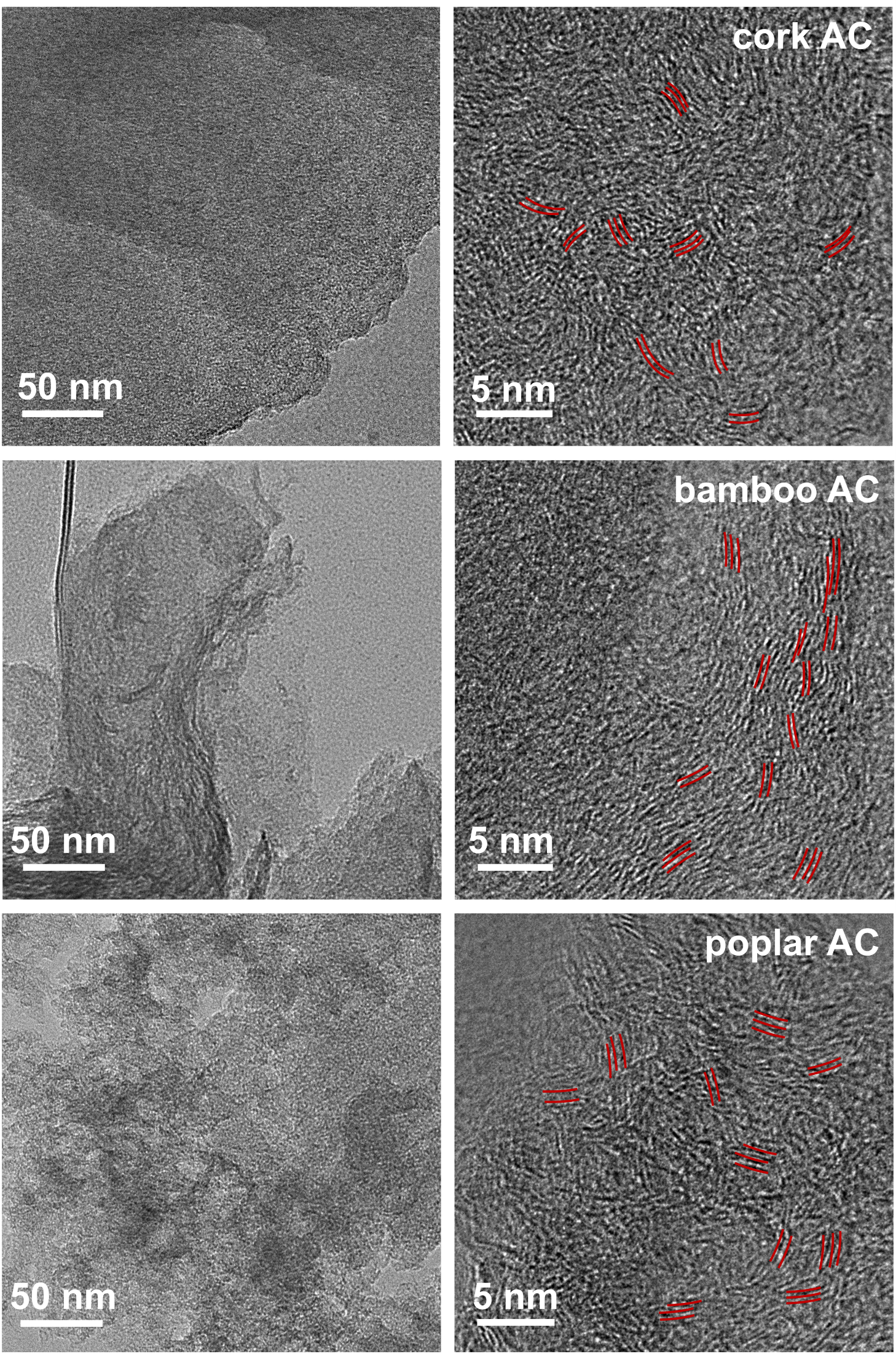
**

**Fig. S16** HR-TEM of other biomass-based ACs with Joule heating


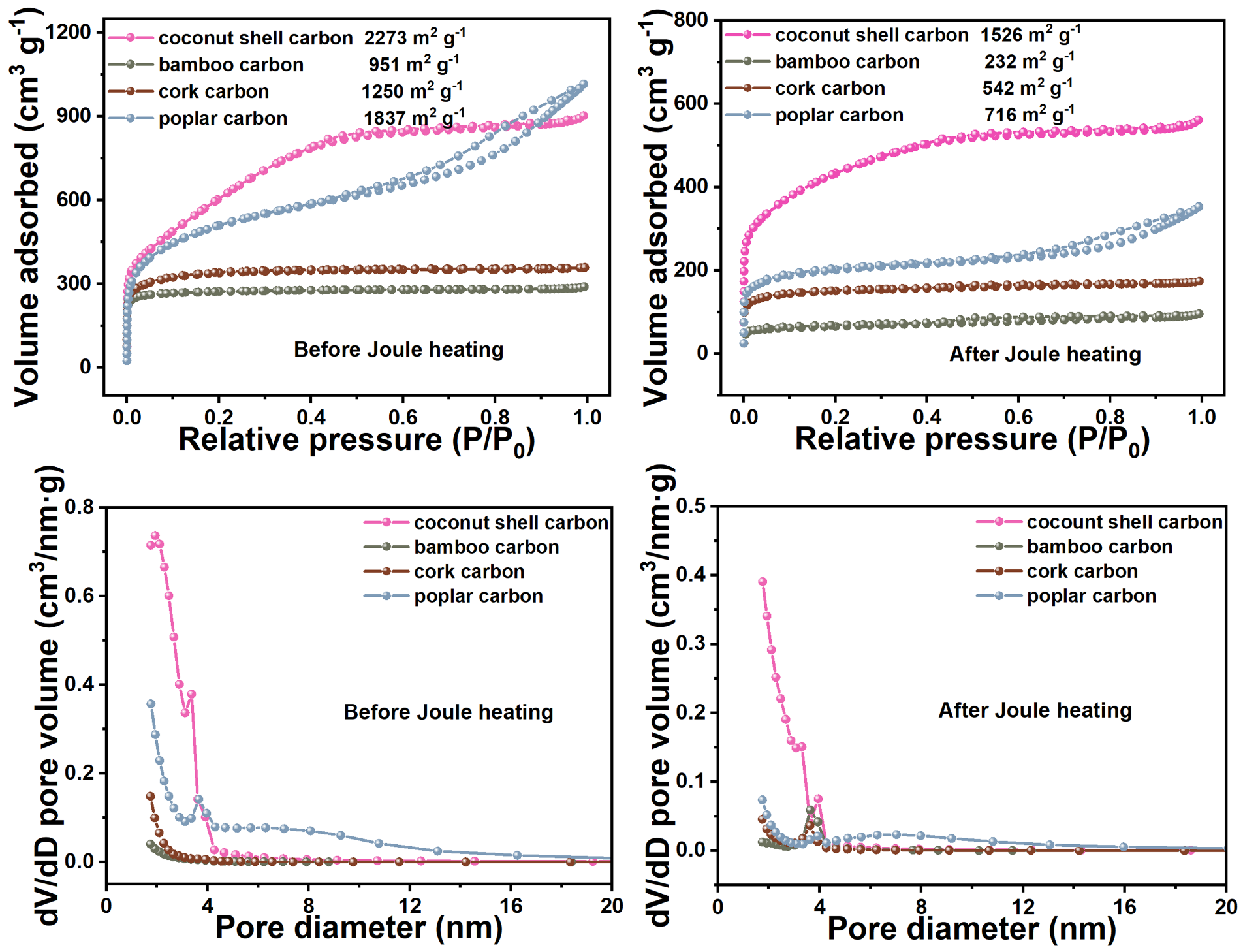


**Fig. S17** N_2_ adsorption–desorption isothermal curves and pore size distribution of biomass-based ACs before and after Joule heating


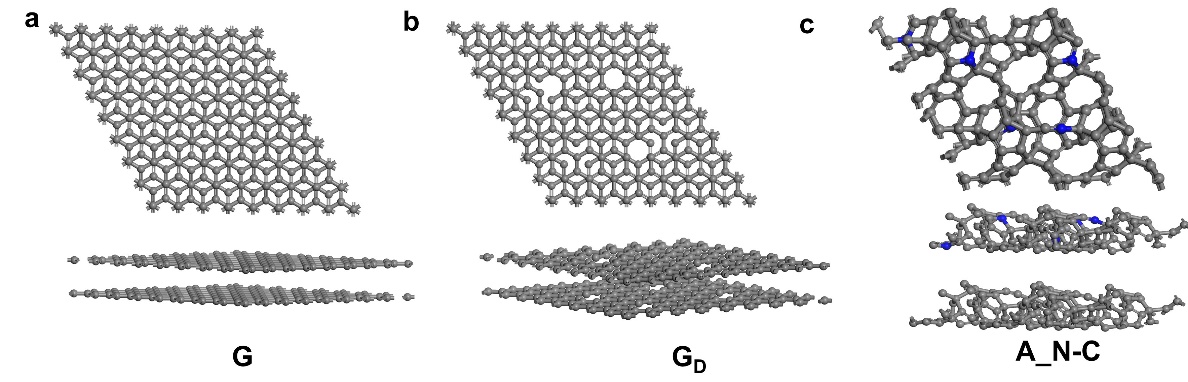


**Fig. S18** DFT calculation model of (**a**) G, (**b**) G_D_, (**c**) A_N-C


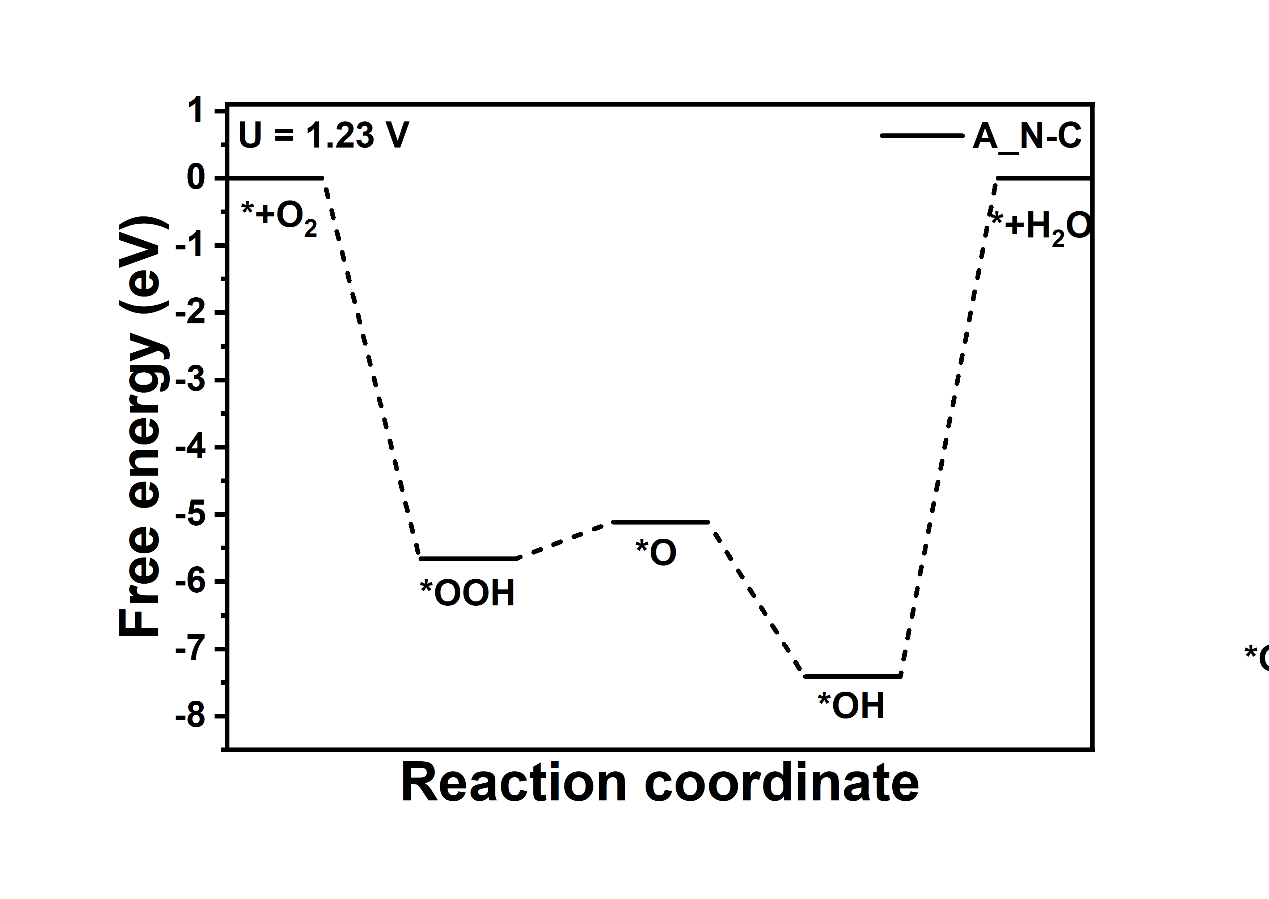


**Fig. S19** Gibbs free energy of the ORR intermediates on A_N-C at 1.23 V


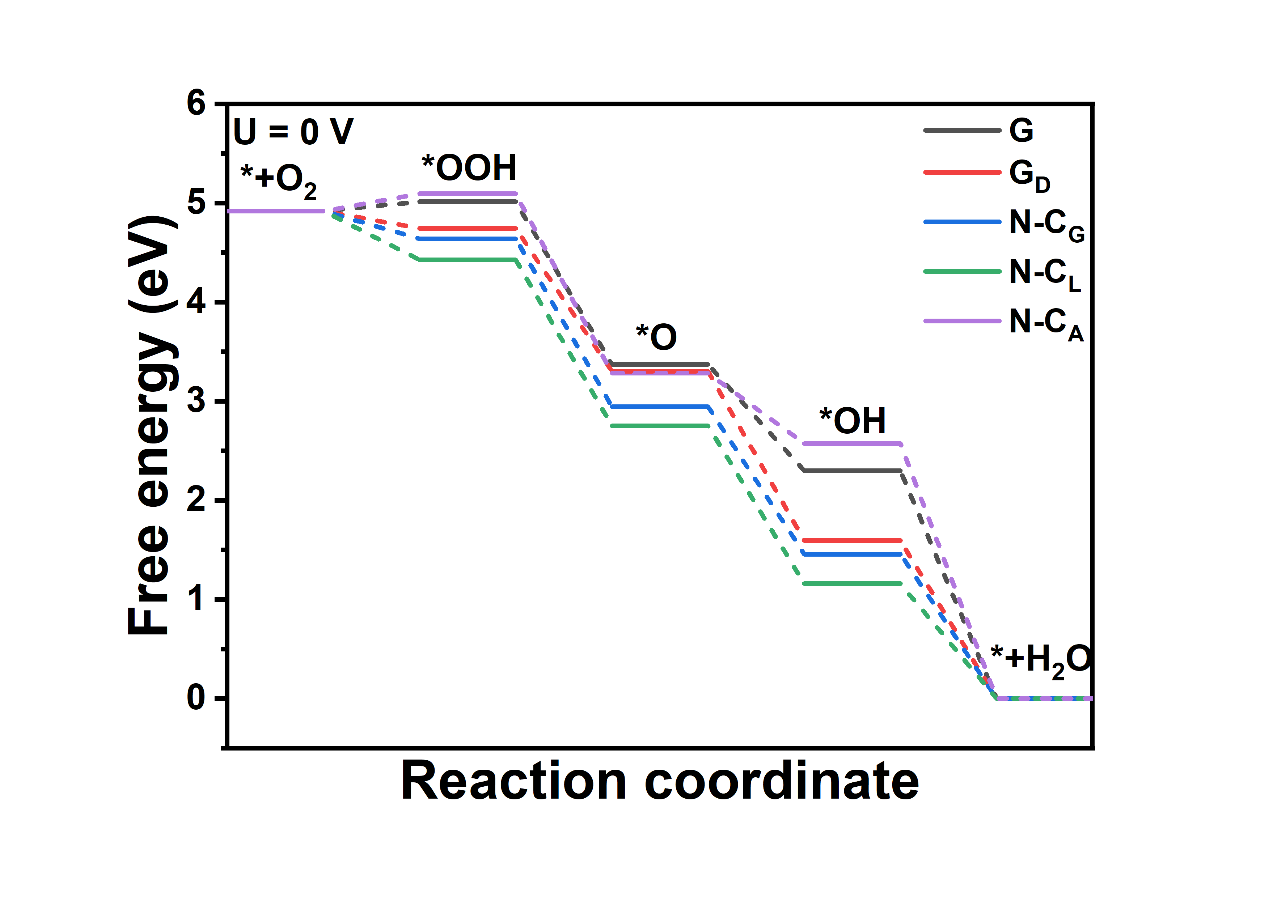


**Fig. S20** Gibbs free energy of the ORR intermediates on different catalysts at 0 V


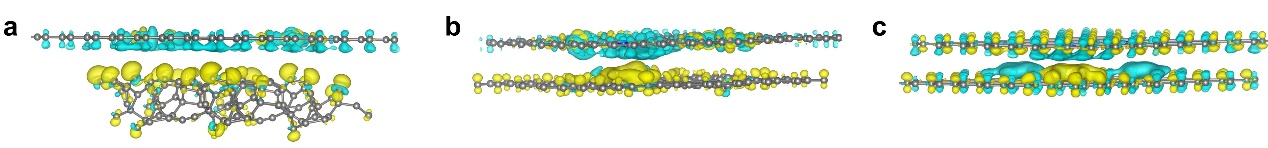


**Fig. S21** (**a-c**) The charge density difference mappings of N-C_A_ (**a**), N-C_L_ (**b**), N-C_G_ (**c**). Yellow: charge accumulation, cyan: charge depletion

**
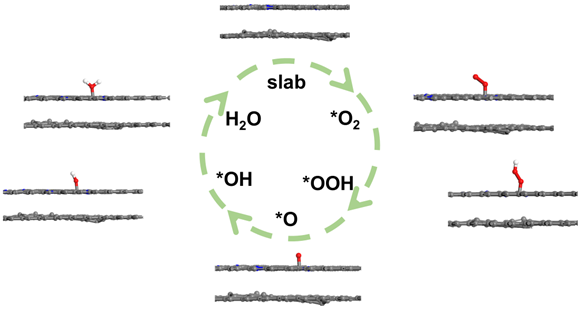
**

**Fig. S22** The optimized adsorption configurations of ORR intermediates (*, *OOH, *O, and *OH) on N-C_L_


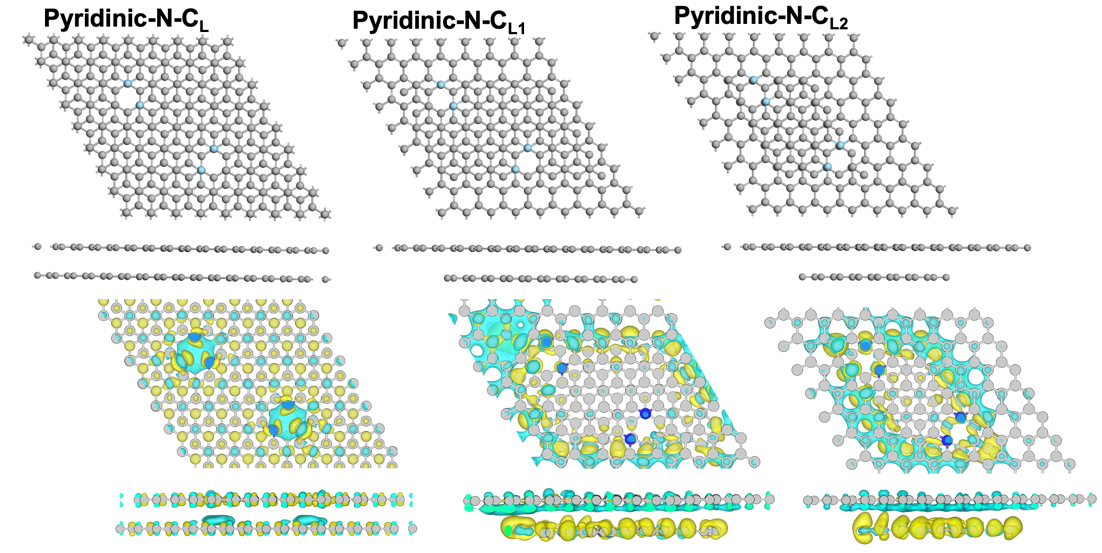

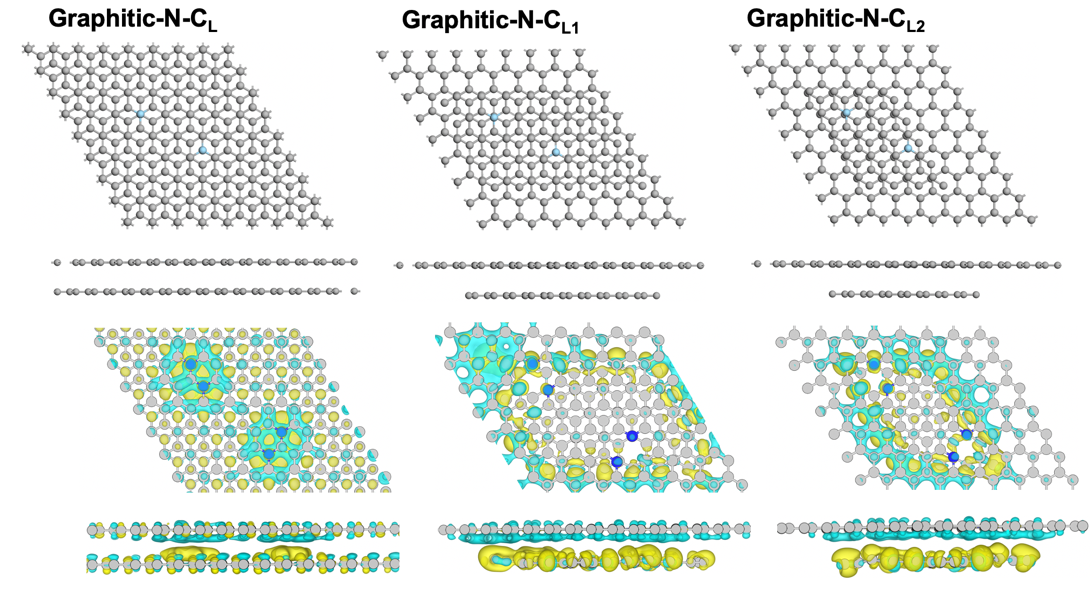


**Fig. S23** The pyridinic N and graphitic N models effect by different size sp^2^-C domains (sp^2^-C size order: C_L_>C_L1_>C_L2_)


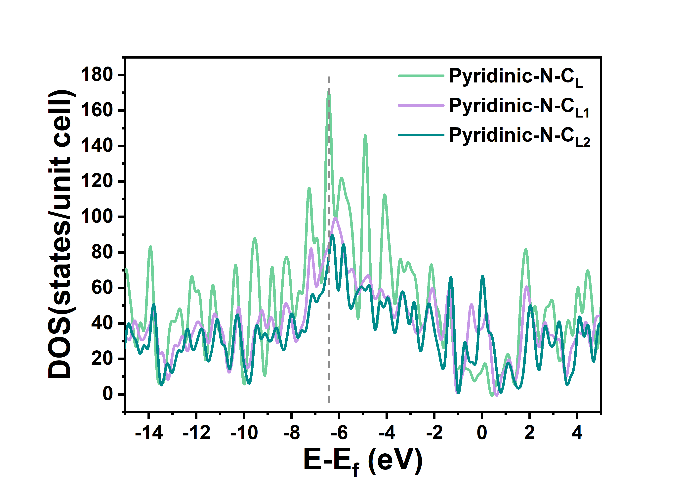

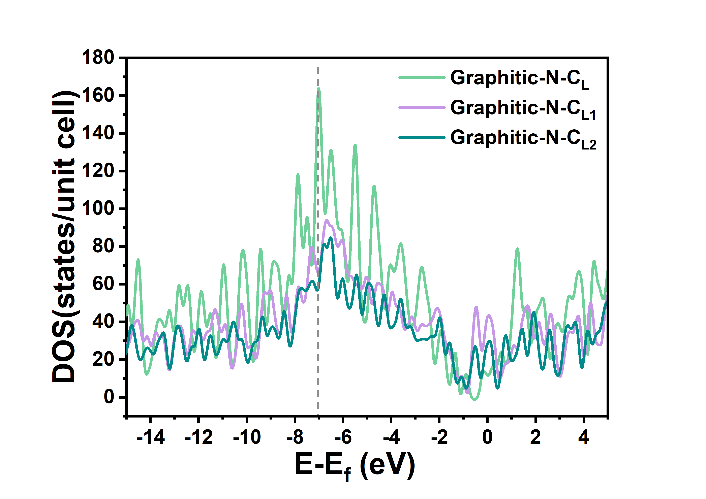


**Fig. S24** The calculated density of states (DOS) of pyridinic N and graphitic N models


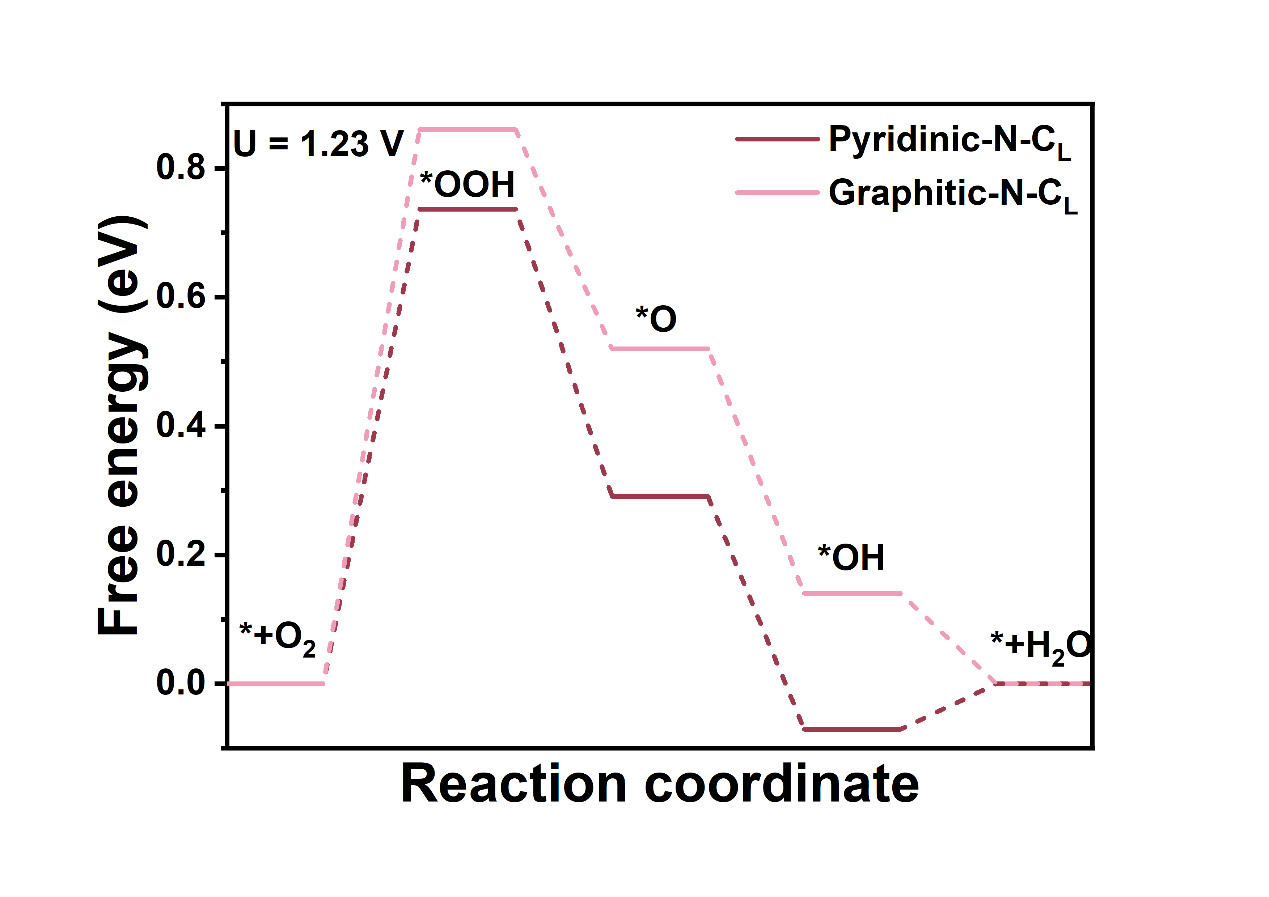


**Fig. S25** Gibbs free energy of the ORR intermediates on pyridinic-N-C_L_ and graphitic-N-C_L_

**
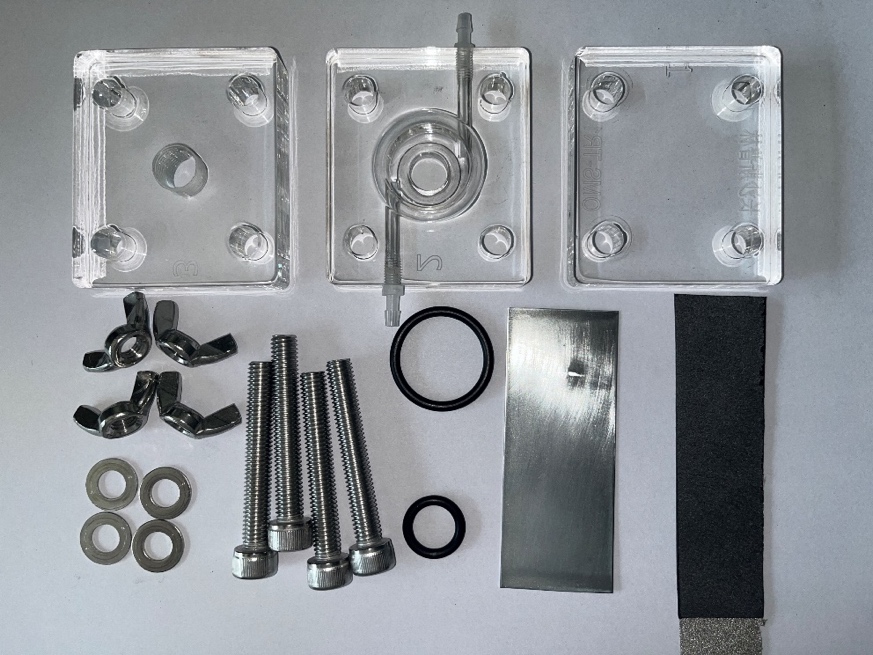
** **
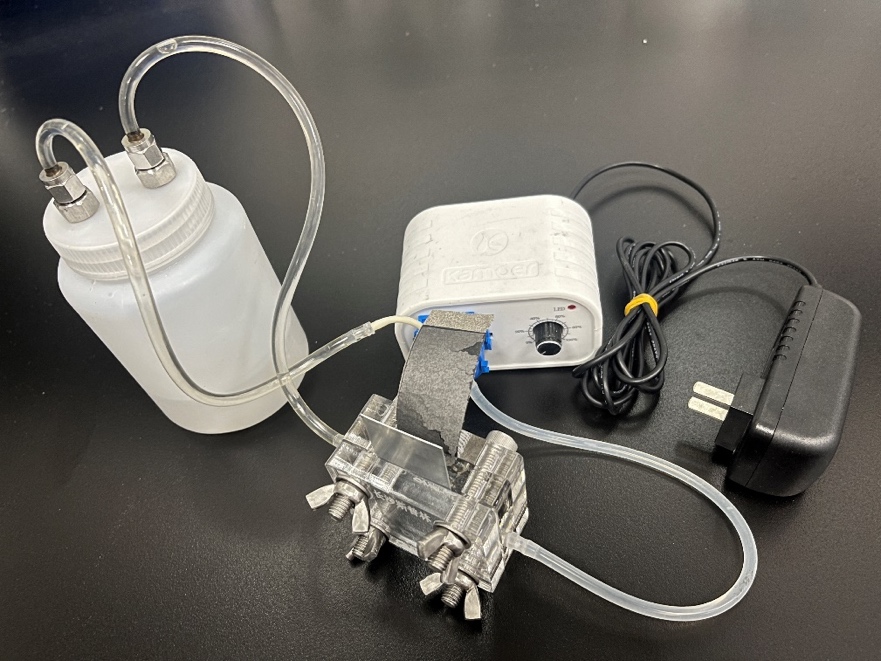
**
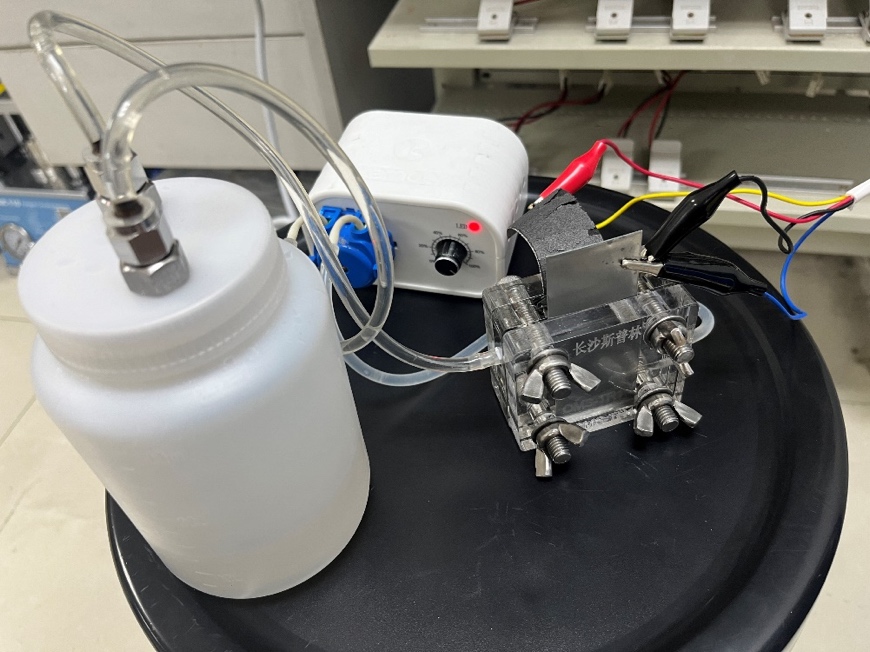


**Fig. S26** Assembled ZAB device

**Table S1** Elemental content of C, N, and O in different catalysts measured by XPS

| Catalysts | C (at%) | N (at%) | O (at%) |
| --- | --- | --- | --- |
| Pure C | 97.88 | / | 2.12 |
| Pure C_D_ | 96.94 | / | 3.06 |
| N-C | 95.94 | 0.89 | 3.17 |
| N-C_D_’ | 92.26 | 1.94 | 5.8 |
| N-C_D_ | 93.03 | 2.35 | 4.62 |

**Table S2** The C, N, O contents of N-C_D_’ treated with a tube furnace

| Catalysts | C (at%) | N (at%) | O (at%) |
| --- | --- | --- | --- |
| N-C_D_ | 93.03 | 2.35 | 4.62 |
| N-C_D_’-1 | 93.19 | 2.22 | 4.58 |
| N-C_D_’-30 | 93.74 | 2.38 | 3.89 |

**Table S3** The comparison with the reported metal-free bifunctional electrocatalysts

| samples | E_ORRonest_  (V) | E_ORR1/2_  (V) | E_OER_(V)  (j=10 mA cm^-2^) | ΔE(V)  (E_j=10_-E_ORR1/2_) | Refs. |
| --- | --- | --- | --- | --- | --- |
| N-C_D_ | 0.98 | 0.884 | 1.525 | 0.64 | This work |
| 20%Pt/C+RuO_2_ | 0.96 | 0.882 | 1.54 | 0.66 | This work |
| 2D-PPCN | 0.92 | 0.85 | 1.595 | 0.75 | [S1] |
| 1100-CNS | 0.99 | 0.85 | 1.69 | 0.84 | [S2] |
| B, N-Carbon | 0.98 | 0.84 | 1.57 | 0.73 | [S3] |
| DG | 0.91 | 0.76 | 1.57 | 0.81 | [S4] |
| GH-BGQD | / | 0.87 | 1.6 | 0.73 | [S5] |
| N-GRW | 0.92 | 0.84 | 1.66 | 0.82 | [S6] |
| NKCNPs | 0.81 | 0.79 | 1.71 | 0.92 | [S7] |
| NPCS-900 | 0.91 | 0.83 | 1.64 | 0.81 | [S8] |
| S, S’-CNT | / | 0.78 | 1.58 | 0.8 | [S9] |
| TTF-F | 0.86 | 0.77 | / | / | [S10] |
| SNGL-20 | 0.86 | 0.68 | / | / | [S11] |
| PG | 0.92 | 0.54 | / | / | [S12] |
| NCNF-1000 | 0.97 | 0.82 | / | / | [S13] |
| NCS-800 | 0.86 | 0.7 | / | / | [S14] |
| MCN-1000-5 | 0.95 | 0.82 | / | / | [S15] |
| G-CBP-A | 0.68 | 0.64 | / | / | [S16] |

**Table S4** The comparison of the performance of assembled Zn-air battery with the reported bifunctional electrocatalysts

| samples | current density(mA cm^-2^) | Time(h) | Refs. |
| --- | --- | --- | --- |
| N-C_D_ | 5 | over 1200 | This work |
| C111-900 | 5 | 200 | [S17] |
| HEO/CoNC | 5 | 140 | [S18] |
| HHPC | 10 | 388 | [S19] |
| N-CNSP | 5 | 150 | [S20] |
| NFS-CNF | 10 | 60 | [S21] |
| N-GDY-900 | 5 | 300 | [S22] |
| NPCTC | 5 | 30 | [S23] |
| PSNC-0.8 | 10 | 320 | [S24] |
| PyN-GDY | 2 | 150 | [S25] |
| SNC | 5 | 500 | [S26] |
| SD-Fe-N/C | 10 | 300 | [S27] |
| WN- Ni@pDC-750–0.02 | 5 | 400 | [S28] |
| NPC-950 | 20 | 85 | [S29] |

**Suppplementary References**

1. W. Lei, Y.-P. Deng, G. Li, Z. P. Cano, X. Wang et al., Two-dimensional phosphorus-doped carbon nanosheets with tunable porosity for oxygen reactions in zinc-air batteries. ACS Catal. **8**, 2464-2472 (2018). <https://doi.org/10.1021/acscatal.7b02739>
2. Z. Pei, H. Li, Y. Huang, Q. Xue, Y. Huang et al., Texturing in situ: N,S-enriched hierarchically porous carbon as a highly active reversible oxygen electrocatalyst. Energy Environ. Sci. **10**, 742-749 (2017). <https://doi.org/10.1039/c6ee03265f>
3. T. Sun, J. Wang, C. Qiu, X. Ling, B. Tian et al., B, N codoped and defect-rich nanocarbon material as a metal-free bifunctional electrocatalyst for oxygen reduction and evolution reactions. Adv. Sci. **5**, 1800036 (2018). <https://doi.org/10.1002/advs.201800036>
4. Y. Jia, L. Zhang, A. Du, G. Gao, J. Chen et al., Defect graphene as a trifunctional catalyst for electrochemical reactions. Adv. Mater. **28**, 9532-9538 (2016). <https://doi.org/10.1002/adma.201602912>
5. T.V. Tam, S.G. Kang, M.H. Kim, S.G. Lee, S.H. Hur et al., Novel graphene hydrogel/b‐doped graphene quantum dots composites as trifunctional electrocatalysts for Zn-air batteries and overall water splitting. Adv. Energy Mater. **9**, 1900945 (2019). <https://doi.org/10.1002/aenm.201900945>
6. H.B. Yang, J. Miao, S.F. Hung, J. Chen, H.B. Tao et al., Identification of catalytic sites for oxygen reduction and oxygen evolution in N-doped graphene materials: Development of highly efficient metal-free bifunctional electrocatalyst. Sci. Adv. **2**, e1501122 (2016). <https://doi.org/10.1126/sciadv.1501122>
7. Q. Wang, Y. Lei, Y. Zhu, H. Wang, J. Feng et al., Edge defect engineering of nitrogen-doped carbon for oxygen electrocatalysts in Zn-air batteries. ACS Appl. Mater. Interf. **10**, 29448-29456 (2018). <https://doi.org/10.1021/acsami.8b07863>
8. S. Chen, L. Zhao, J. Ma, Y. Wang, L. Dai et al., Edge-doping modulation of N, P-codoped porous carbon spheres for high-performance rechargeable Zn-air batteries. Nano Energy **60**, 536-544 (2019). <https://doi.org/10.1016/j.nanoen.2019.03.084>
9. A. M. El‐Sawy, I. M. Mosa, D. Su, C. J. Guild, S. Khalid et al., Controlling the active sites of sulfur‐doped carbon nanotube-graphene nanolobes for highly efficient oxygen evolution and reduction catalysis. Adv. Energy Mater. **6**, 1501966 (2015). <https://doi.org/10.1002/aenm.201501966>
10. L. Hao, S. Zhang, R. Liu, J. Ning, G. Zhang et al., Bottom-up construction of triazine-based frameworks as metal-free electrocatalysts for oxygen reduction reaction. Adv. Mater. **27**, 3190-3195 (2015). <https://doi.org/10.1002/adma.201500863>
11. J. Xu, G. Dong, C. Jin, M. Huang, L. Guan, Sulfur and nitrogen co-doped, few-layered graphene oxide as a highly efficient electrocatalyst for the oxygen-reduction reaction. ChemSusChem **6**, 493-499 (2013). <https://doi.org/10.1002/cssc.201200564>
12. C. Zhang, N. Mahmood, H. Yin, F. Liu, Y. Hou, Synthesis of phosphorus-doped graphene and its multifunctional applications for oxygen reduction reaction and lithium ion batteries. Adv. Mater. **25**, 4932-4937 (2013). <https://doi.org/10.1002/adma.201301870>
13. Q. Liu, Y. Wang, L. Dai, J. Yao, Scalable fabrication of nanoporous carbon fiber films as bifunctional catalytic electrodes for flexible Zn-air batteries. Adv. Mater. **28**, 3000-3006 (2016). <https://doi.org/10.1002/adma.201506112>
14. P. Chen, L.-K. Wang, G. Wang, M.-R. Gao, J. Ge et al., Nitrogen-doped nanoporous carbon nanosheets derived from plant biomass: an efficient catalyst for oxygen reduction reaction. Energy Environ. Sci. **7**, 4095-4103 (2014). <https://doi.org/10.1039/c4ee02531h>
15. H. Jiang, J. Gu, X. Zheng, M. Liu, X. Qiu et al., Defect-rich and ultrathin N doped carbon nanosheets as advanced trifunctional metal-free electrocatalysts for the ORR, OER and HER. Energy Environ. Sci. **12**, 322-333 (2019). <https://doi.org/10.1039/c8ee03276a>
16. Y. Zhang, X. Zhuang, Y. Su, F. Zhang, X. Feng, Polyaniline nanosheet derived B/N co-doped carbon nanosheets as efficient metal-free catalysts for oxygen reduction reaction. J. Mater. Chem. A **2**, 7742-7746 (2014). <https://doi.org/10.1039/c4ta00814f>
17. Y. Zhou, J. Wu, Z. Wang, H. Huang, Y. Liu et al., A biomass derived porous carbon materials with adjustable interfacial electron transmission dynamics as highly-efficient air cathode for Zn-Air battery. Mater. Res. Bull. **153**, 111908 (2022). <https://doi.org/10.1016/j.materresbull.2022.111908>
18. T. Yu, H. Xu, Z. Jin, Y. Zhang, H.-J. Qiu, Noble metal-free high-entropy oxide/Co-N-C bifunctional electrocatalyst enables highly reversible and durable Zn-air batteries. Appl. Surf. Sci. **610**, 155624 (2023). <https://doi.org/10.1016/j.apsusc.2022.155624>
19. X. Xiao, X. Li, Z. Wang, G. Yan, H. Guo et al., Robust template-activator cooperated pyrolysis enabling hierarchically porous honeycombed defective carbon as highly-efficient metal-free bifunctional electrocatalyst for Zn-air batteries. Appl. Catal. B Environ. **265**, 118603 (2020). <https://doi.org/10.1016/j.apcatb.2020.118603>
20. L. Zong, W. Wu, S. Liu, H. Yin, Y. Chen et al., Metal-free, active nitrogen-enriched, efficient bifunctional oxygen electrocatalyst for ultrastable zinc-air batteries. Energy Storage Mater. **27**, 514-521 (2020). <https://doi.org/10.1016/j.ensm.2019.12.013>
21. H. Li, T. A. Ha, S. Jiang, C. Pozo-Gonzalo, X. Wang et al., N, F and S doped carbon nanofibers generated from electrospun polymerized ionic liquids for metal-free bifunctional oxygen electrocatalysis. Electrochim. Acta **377**, 138089 (2021). <https://doi.org/10.1016/j.electacta.2021.138089>
22. T. Lu, X. Hu, J. He, R. Li, J. Gao et al., Aqueous/solid state Zn-air batteries based on N doped graphdiyne as efficient metal-free bifunctional catalyst. Nano Energy **85**, 106024 (2021). <https://doi.org/10.1016/j.nanoen.2021.106024>
23. Y. Li, Z. Yan, Q. Wang, H. Ye, M. Li et al., Ultrathin, highly branched carbon nanotube cluster with outstanding oxygen electrocatalytic performance. Electrochim. Acta **282**, 224-232 (2018). https://doi.org/10.1016/j.electacta.2018.06.058
24. X. Xiao, H. Zhao, L.-F. Li, B.-L. Qu, Y.-L. Wu et al., Ion exchange coupled biomineral self-sacrificial template synthesis of N-enriched porous carbon as robust electrocatalyst for rechargeable Zn-air battery. Rare Metals **42**, 1186-1194 (2023). <https://doi.org/10.1007/s12598-022-02190-z>
25. Q. Lv, N. Wang, W. Si, Z. Hou, X. Li et al., Pyridinic nitrogen exclusively doped carbon materials as efficient oxygen reduction electrocatalysts for Zn-air batteries. Appl. Catal. B Environ. **261**, 118234 (2020). <https://doi.org/10.1016/j.apcatb.2019.118234>
26. Y. Guo, S. Yao, L. Gao, A. Chen, M. Jiao et al., Boosting bifunctional electrocatalytic activity in S and N co-doped carbon nanosheets for high-efficiency Zn-air batteries. J. Mater. Chem. A **8**, 4386-4395 (2020). <https://doi.org/10.1039/c9ta12762c>
27. J.-C. Li, Y. Meng, H. Zhong, L. Zhang, S. Ding et al., Supramolecular complex derived carbon nanotubes decorated with iron single atoms and nanoclusters as efficient bifunctional oxygen electrocatalysts for rechargeable Zn-air batteries. Carbon **205**, 302-309 (2023). <https://doi.org/10.1016/j.carbon.2023.01.024>
28. Y. Du, W. Chen, Z. Zhong, S. Wang, L. Zhou et al., Bifunctional oxygen electrocatalysts with WN@Ni nanostructures implanted on N-doped carbon nanorods for rechargeable Zn-Air batteries. J. Alloy Compd. **960**, 170789 (2023). <https://doi.org/10.1016/j.jallcom.2023.170789>
29. Z. Li, S. Ji, H. Liu, C. Xu, C. Guo et al., Constructing asymmetrical coordination microenvironment with phosphorus‐incorporated nitrogen‐doped carbon to boost bifunctional oxygen electrocatalytic activity. Adv. Funct. Mater. **34**, 2314444 (2024). <https://doi.org/10.1002/adfm.202314444>
